# Characterization and Identification of the Ent-Kaurane Diterpenoids in Isodonis Excisoidis Herba Using UHPLC-LTQ-Orbitrap-MS

**DOI:** 10.3390/molecules31020317

**Published:** 2026-01-16

**Authors:** Xiaoya Sun, Lingxia Zhang, Conglong Lian, Suiqing Chen, Liping Dai, Yaozu Han

**Affiliations:** 1 Collaborative Innovation Center of Research and Development on the Whole Industry Chain of Yu-Yao, Henan University of Chinese Medicine, No. 156, Jinshui East Road, Zhengzhou 450046, China; sxy7336@163.com (X.S.); zhanglingxia1205@126.com (L.Z.); 2 School of Pharmacy, Henan University of Chinese Medicine, No. 156, Jinshui East Road, Zhengzhou 450046, China; liancl00@163.com (C.L.); 15537904030@163.com (Y.H.); 3 Henan Lingrui Pharmaceutical Company Limited, Jiangjun Road, Xinxian, Xinyang 465550, China; 4 Collaborative Innovation Center for Respiratory Disease Diagnosis and Treatment & Chinese Medicine Development of Henan Province, Henan University of Chinese Medicine, No. 156, Jinshui East Road, Zhengzhou 450046, China

**Keywords:** Isodonis Excisoidis Herba, UHPLC-LTQ-Orbitrap-MS, chemical constituents, identification, diagnostic fragmentation

## Abstract

Isodonis Excisoidis Herba (IEH) is a newly discovered herbal medicine used to treat esophageal cancer, chronic pharyngitis, and hepatitis, and ent-kaurane diterpenoids are its main active components. However, systematic studies on the chemical profile of ent-kaurane diterpenoids are lacking. In this study, UHPLC-LTQ-Orbitrap-MS was performed to investigate the fragmentation behaviors of three different types of ent-kaurane diterpenoids from IEH. Bridgehead-unsubstituted 7,20-epoxy-ent-kaurane diterpenoids yielded ions with typical losses of R_7_H, R_1_H, R_14_H, CH_2_O, CO, and R_6_H. The [M + NH_4_ − NH_3_ − R_20_]^+^ precursor ions at 331.1895 and the characteristic ions at *m*/*z* 313.1792, 295.1686, 285.1842, 277.1581, 267.1737, and 249.1632 were the most possible fragmentation pathways for bridgehead-substituted 7,20-epoxy-ent-kaurane diterpenoids. Fragmentation with the successive loss of multiple 18, 42, or 60 Da occurring in the OH groups and OAc groups is characteristic of 7,20-non-epoxy-kaurane diterpenoids. Using accurate mass measurements for each precursor ion and the subsequent fragmented ions, a total of 94 ent-kaurane diterpenoids were identified or tentatively characterized in IEH, including 48 potentially new ent-kaurane diterpenoids.

## 1. Introduction

*Isodon excisoides* (Y. Z. Sun ex C. H. Hu) H. Hara (IE) is a perennial medicinal herb belonging to the genus *Isodon* in the Labiatae family, mainly distributed in the western region of Henan and Yunnan Provinces in China [1]. Isodonis Excisoidis Herba (the aerial parts of IE, IEH) has been listed in the Chinese Materia Medica and prescribed for the treatment of cold headaches, rheumatic arthralgia, bruises, fractures, bleeding wounds, and snake bites [2]. Meanwhile, local residents also use it to make tea, decoctions, and dressings for treating esophageal cancer, chronic pharyngitis, and hepatitis [3].

Ent-kaurane diterpenoids are the primary bioactive constituents of *Isodon* plants [4]. For instance, the highly anticipated oridonin possesses promising anti-tumor [5] and anti-leukemia properties [6] and serves as a covalent NLRP3 inhibitor with strong anti-inflammasome activity [7]. Notably, several ent-kaurane diterpenoids with anti-tumor activity have been isolated from *Isodon excisoides* [8,9,10,11,12,13,14]. Consistent with these reports, our laboratory has isolated and identified 12 ent-kaurane diterpenoids from IEH, including oridonin and lasiokaurin, confirming their significant anti-tumor activity [8,9,10]. However, the structures of ent-kaurane diterpenoids are diverse and mostly homologous, making it difficult to separate and purify them through conventional chemical composition separation methods. In particular, the trace components cannot be enriched and identified, substantially restricting research on the anti-tumor substance basis of this plant.

With the rapid development of chromatography and mass spectrometry (MS) techniques, ultra-high-performance liquid chromatography coupled with electrospray ionization hybrid linear trap quadrupole orbitrap mass spectrometry (UHPLC-LTQ-Orbitrap-MS) has become an extremely powerful tool for the targeted and untargeted profiling of natural products from complex matrices [15]. Compared with low-resolution mass spectrometry methods, LTQ-Orbitrap-MS not only provides high sensitivity with a mass accuracy of less than 10 ppm but also offers an unequivocal assignment of the product ions to their respective precursor ions in MS*^n^* (*n* ≥ 3) experiments, allowing us to precisely describe the fragmentation pathways [16]. However, to our knowledge, only a limited number of studies have employed liquid chromatography–mass spectrometry (LC-MS) to analyze *Isodon* species or their isolated ent-kaurane diterpenoid monomers, with reports on *Isodon rubescens* [17,18,19], *Isodon serra* [20,21], *Isodon japonica* [22], *Rabdosia rubescens* [23,24], and oridonin [25,26]. Furthermore, the existing literature primarily concentrates on the scanning and analysis of ent-kaurane diterpenoids in the negative ion mode [19,21,22,23,25,26] or on the quantitative analysis of known monomers from *Isodon* species plants in the positive mode [17,18,20,24]. Research dedicated to elucidating their fragmentation pathways in the positive ion mode remains scarce. Therefore, systematically analyzing multiple ent-kaurane diterpenoids using this highly sensitive and rapid method in the positive ion mode is of significant value. In this study, we focus on a comprehensive study of the MS*^n^* spectra on an LTQ-Orbitrap-MS to elucidate the fragmentation behavior of 12 known compounds isolated from IEH, and we also apply characteristic fragmentation patterns to preliminarily identify additional unknown chemical constituents from this herbal medicine. In this study, twelve compounds were divided into three types according to their basic skeletons: bridgehead-unsubstituted 7,20-epoxy-ent-kaurane diterpenoids, bridgehead-substituted 7,20-epoxy-ent-kaurane diterpenoids, and 7,20-non-epoxy-kaurane diterpenoids. Based on the fragmentation patterns, accurate mass measurements, and retention times, a total of 94 compounds were unambiguously identified or tentatively characterized, 48 of which are potentially new ent-kaurane diterpenoids. This is the first determination of ent-kaurane diterpenoids in IEH using UHPLC-LTQ-Orbitrap-MS in the positive ion mode.

## 2. Results and Discussion

### 2.1. Optimization of the LC and MS Conditions

The UHPLC-LTQ-Orbitrap-MS conditions were systemically optimized to improve the resolution and sensitivity of the analysis and obtain appropriate ionization. Key UHPLC parameters, including the chromatographic column (Syncronis-C18, ACQUITY UPLC HSS-T3, and ACQUITY UPLC BEH-C18), mobile phase compositions (deionized water, deionized water containing 0.1% formic acid, and acetonitrile), gradient profile, and flow rate (0.2–0.5 mL/min), were evaluated and refined with peak resolution as the primary criterion. The results indicated that the ACQUITY UPLC BEH-C18 column with a small particle size (1.7 μm) provided better separation with a higher peak resolution, and that a mobile phase consisting of deionized water with 0.1% formic acid and aqueous acetonitrile outperformed the others.

For LTQ-Orbitrap-MS conditions optimization, positive ion mode was selected due to the stronger signal response observed for the twelve known ent-kaurane diterpenoids and other sample components compared with the negative ion mode. The mass spectrometric parameters were initially tuned by direct infusion of reference standards, employing a resolution power of 30,000 in full-scan mode followed by MS*^n^* scan in the Orbitrap (also at R = 30,000) with a 10 s exclusion duration. The collision energies for MS^2^ and MS^3^ were optimized over a range up to 35% of the maximum collision-induced dissociation (CID) energy to yield informative fragment ion profiles. It was observed that at the initial test level of 35%, fragment ions in the higher *m*/*z* range showed relatively low abundance, complicating the determination of stepwise fragmentation pathways. Therefore, the CID energies were adjusted to levels that produced more informative profiles suitable for pathway elucidation, ultimately set to 25% for MS^2^ and 20% for MS^3^ of the maximum CID energy, thereby ensuring appropriate ionization and informative MS abundance data in the positive ion mode.

### 2.2. Identification of the Ent-Kaurane Diterpenoids of IEH

**Figure 1:**
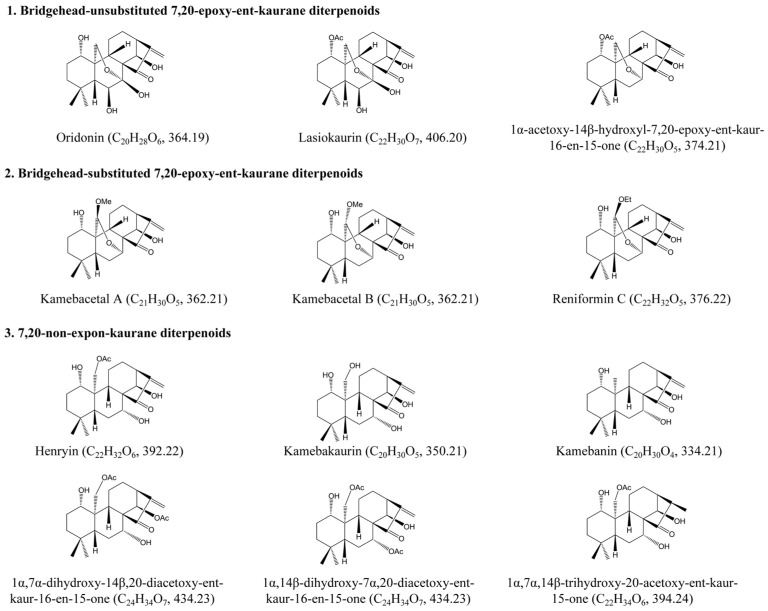
Chemical structures of the three different types of ent-kaurane diterpenoids identified from Isodonis Excisoidis Herba.

**Figure 2:**
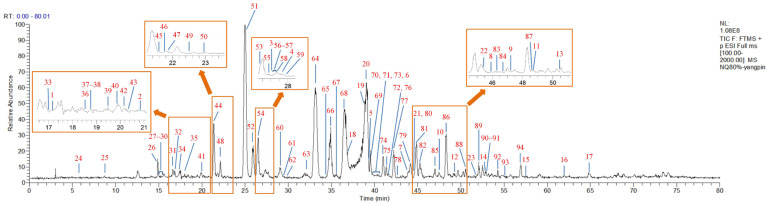
Total ion chromatogram (TIC) of Isodonis Excisoidis Herba in the positive ion mode using UHPLC-LTQ-Orbitrap-MS. The peak number corresponds to the compound number in Table 2 and Supplementary Table S1.

Based on the structural characteristics of the twelve reference compounds (Figure 1), they were divided into three groups: bridgehead-unsubstituted 7,20-epoxy-ent-kaurane diterpenoids, including oridonin (**2**), lasiokaurin (**5**), and 1α-acetoxy-14β-hydroxyl-7,20-epoxy-ent-kaur-16-en-15-one (**13**); bridgehead-substituted 7,20-epoxy-ent-kaurane diterpenoids, including kamebacetal A (**21**), kamebacetal B (**22**), and reniformin C (**23**); and 7,20-non-epoxy-kaurane diterpenoids, consisting of kamebakaurin (**44**), kamebanin (**52**), 1α,7α,14β-trihydroxy-20-acetoxy-ent-kaur-15-one (**54**), henryin (**57**), 1α,7α-dihydroxy-14β,20-diacetoxy-ent-kaur-16-en-15-one (**64**), and 1α,14β-dihydroxy-7α,20-diacetoxy-ent-kaur-16-en-15-one (**74**). The fragmentation patterns and pathways of these reference compounds are shown in Table 1. The confirmation of known compounds in IEH for which reference standards were available was performed based on coincidence in retention time, precursor ions, fragment ions, and their abundance patterns with respect to the reference standards analyzed under identical LC-MS conditions. Moreover, the fragmentation patterns and pathways of the standards were investigated in depth to further confirm the tentative structures of their derivatives. For the unavailable standard compounds, the tentative structures were proposed based on the following steps: (1) The molecular formula was established based on high-accuracy protonated precursors, such as [M + H]^+^, [M + NH_4_]^+^, and [M + H − CH_4_O]^+^, within a mass error of 10 ppm and the fractional isotope abundance. (2) When there were several matching isomers, the structures were resolved by querying the chemical information database of IEH components—which was compiled from previously reported compounds in *Isodon* species, particularly IEH [8,9,10,11,12,17,18,19,20,21,22,23,24,25,26,27]—and by comparing their distinct retention times in the total ion chromatogram (TIC), along with characteristic fragment ion abundance patterns. (3) The fragment ions from MS*^n^* mass spectrometry, together with the fragmentation pathways of known compounds and theoretical simulations from Thermo Scientific™ Mass Frontier 7.0 software, were integrated to propose tentative structures. In particular, for the 7,20-non-epoxy-kaurane diterpenoids, kamebakaurin, henryin, etc., the fragmentation rules for the standards were applied to elucidate the tentative structures of the corresponding derivatives with the same basic skeleton. The total ion chromatograms (TICs) of the IEH samples were acquired in the positive mode for further confirmation (shown in Figure 2). Based on the MS*^n^* analysis and accurate mass measurements and retention times, a total of 94 components, including 17 bridgehead-unsubstituted 7,20-epoxy-ent-kaurane diterpenoids, 6 bridgehead-substituted 7,20-epoxy-ent-kaurane diterpenoids, and 71 7,20-non-epoxy-kaurane diterpenoids, were unambiguously or tentatively identified from IEH. The key diagnostic ions and fragmentation pathways are summarized in Table 2, and the complete MS*^n^* dataset is provided in the Supplementary Materials (Table S1).

#### 2.2.1. Bridgehead-Unsubstituted 7,20-Epoxy-Ent-Kaurane Diterpenoids

**Figure 3:**
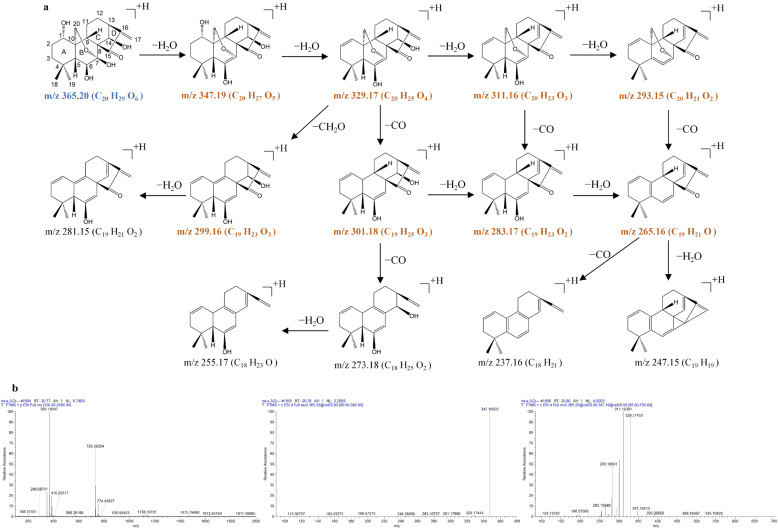
The fragmentation pathway of oridonin recorded using a collision energy of 25 V (**a**) and its CID MS/MS spectrum (**b**).

**Figure 4:**
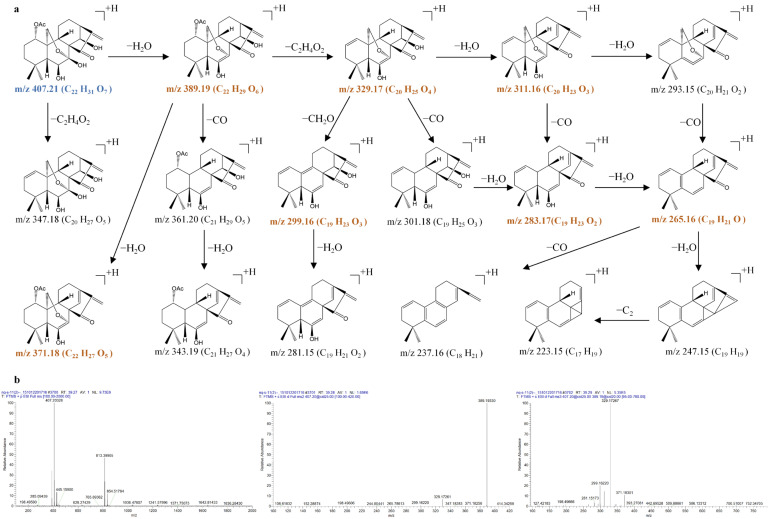
The fragmentation pathway of lasiokaurin recorded using a collision energy of 25 V (**a**) and its CID MS/MS spectrum (**b**).

**Figure 5:**
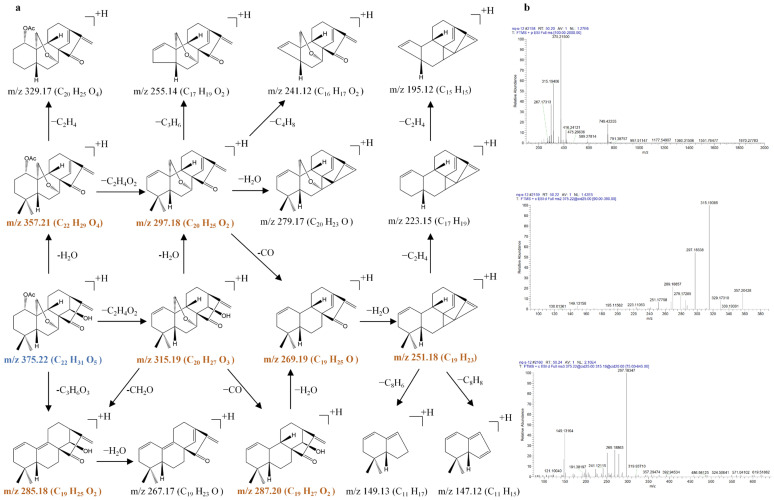
The fragmentation pathway of 1α-acetoxy-14β-hydroxyl-7,20-epoxy-ent-kaur-16-en-15-one using a collision energy of 25 V (**a**) and its CID MS/MS spectrum (**b**).

In the present study, three reference compounds—oridonin (**2**), lasiokaurin (**5**), and 1α-acetoxy-14β-hydroxyl-7,20-epoxy-ent-kaur-16-en-15-one (**13**)—were first introduced in the positive ion modes of the ESI source. Because of their remarkably higher abundance in the positive ion mode, the [M + H]^+^ or [M + NH_4_]^+^ ions were selected as the precursor ions for CID fragmentation to produce the MS/MS spectra. Then, the prominent MS/MS product ions were selected for further MS*^n^* analysis (*n* = 3–4). The fragmentation pathways and MS*^n^* mass spectra of oridonin (**2**), lasiokaurin (**5**), and 1α-acetoxy-14β-hydroxyl-7,20-epoxy-ent-kaur-16-en-15-one (**13**) are shown in Figure 3, Figure 4 and Figure 5.

Oridonin (**2**) exhibits the [M + H]^+^ ion at *m*/*z* 365.1955 (C_20_H_29_O_6_) and the [2M + H]^+^ ion at *m*/*z* 729.3835 (C_40_H_57_O_12_) in the MS^1^ spectra. The [M + H]^+^ ion at *m*/*z* 365.1955 (C_20_H_29_O_6_) produced a prominent product ion at *m*/*z* 347.1850 (C_20_H_28_O_5_) through the typical loss of H_2_O. The ion at *m*/*z* 347.1850 was further fragmented to produce the prominent ions at *m*/*z* 329.1745 (C_20_H_25_O_4_), 311.1639 (C_20_H_23_O_3_), 283.1690 (C_19_H_23_O_2_), and 265.1585 (C_19_H_21_O) by typical losses of 18 Da (H_2_O), 18 Da (H_2_O), 28 Da (CO), and 18 Da (H_2_O) in the MS^3^ spectrum. Notably, the loss of CO was apparent at *m*/*z* 329.1745 (*m*/*z* 329.1745 → 301.1796), *m*/*z* 311.1639 (*m*/*z* 311.1639 → 283.1690), and *m*/*z* 293.1535 (*m*/*z* 293.1535 → 265.1585), and the loss of CH_2_O was apparent at *m*/*z* 329.1745 (*m*/*z* 329.1745 → 299.1639) in the MS^3^ spectra, suggesting that the methyleneoxy between C-7 and C-10 was rearranged on the B-ring. The loss of H_2_O was also apparent in the MS^3^ spectra at *m*/*z* 347.1850 (*m*/*z* 347.1850 → 329.1745), *m*/*z* 329.1745 (*m*/*z* 329.1745 → 311.1639), *m*/*z* 311.1639 (*m*/*z* 311.1639 → 293.1535), and *m*/*z* 283.1690 (*m*/*z* 283.1690 → 265.1585), suggesting the cleavage of the hydroxyl groups of oridonin. Due to the existence of the anacetoxy group at C-1, the characteristic fragment ions of lasiokaurin (**5**) were *m*/*z* 407.2052 (C_22_H_31_O_7_), *m*/*z* 389.1949 (C_22_H_29_O_6_), and *m*/*z* 371.1843 (C_22_H_27_O_5_). The characteristic ions of lasiokaurin (**5**) were 42 Da (C_2_H_2_O) larger than those of oridonin. The other ions at *m*/*z* 329.1739 (C_20_H_25_O_4_), 311.1634 (C_20_H_23_O_3_), 299.1634 (C_19_H_23_O_3_), 283.1684 (C_19_H_23_O_2_), and 265.1578 (C_19_H_21_O) were from the fragment ions at *m*/*z* 389.1949 (C_22_H_29_O_6_, [M + H‒H_2_O]^+^) via typical losses of 60 Da (C_2_H_4_O_2_), 18 Da (H_2_O), 30 Da (CH_2_O), 28 Da (CO), and 18 Da (H_2_O) in the fragmentation pathways.

Compounds **1**, **4**, and **6** are oridonin derivatives, and they showed similar fragmentation behaviors as oridonin (**2**). Compound **1** generated the same [M + H]^+^ ions at *m*/*z* 365.1951 (C_20_H_29_O_6_) and the [M + NH_4_]^+^ ion at *m*/*z* 382.2213 (C_20_H_32_O_6_N) in the positive mode, and a dominant ion is generated at *m*/*z* 347.1846 (C_20_H_28_O, [M + NH_4_ − NH_3_ − H_2_O]^+^) in the MS^3^ spectrum. Therefore, it is tentatively identified as an isomer of oridonin. Compound **4** generated [M + H]^+^ precursor ions at *m*/*z* 349.2002 (C_20_H_29_O_5_), which displayed common fragment ions at *m*/*z* 331.1897 (C_20_H_27_O_4_), 313.1792 (C_20_H_25_O_3_), 295.1686 (C_20_H_23_O_2_), 285.1844 (C_19_H_25_O_2_), 267.1736 (C_19_H_23_O), and *m*/*z* 249.1617 (C_19_H_21_). All were 16 Da smaller than the characteristic ions of oridonin (**2**), suggesting that Compound **4** represents oridonin (**2**) lacking OH elements. Compound **6** had [M + H]^+^ ions at *m*/*z* 347.1845 (C_20_H_27_O_5_) and the [M + NH_4_]^+^ ion at *m*/*z* 364.2108 (C_20_H_30_O_5_N), and it had fragment ions at *m*/*z* 329.1740 (C_20_H_25_O_4_), 311.1631 (C_20_H_23_O_3_), 283.1688 (C_19_H_23_O_2_), and 265.1581 (C_19_H_21_O). The characteristic ions of Compound **6** were 18 Da (H_2_O) smaller than oridonin, suggesting that it represents the reference oridonin compound lacking one hydroxyl group and a double bond addition.

1α-acetoxy-14β-hydroxyl-7,20-epoxy-ent-kaur-16-en-15-one (**13**) produced the [M + H]^+^ ion at *m*/*z* 375.2150 (C_22_H_31_O_5_) and the [2M + H]^+^ ion at *m*/*z* 749.4241 (C_44_H_61_O_10_) in the MS^1^ spectra. The [M + H]^+^ ion at *m*/*z* 375.2150 (C_22_H_31_O_5_) produced prominent product ions at *m*/*z* 357.2051 (C_22_H_29_O_4_) through a typical loss of H_2_O and at *m*/*z* 315.1946 (C_20_H_27_O_3_) through a typical loss of C_2_H_4_O_2_. The ion at *m*/*z* 315.1946 was further fragmented to produce prominent ions at *m*/*z* 297.1839 (C_20_H_25_O_2_), 269.1890 (C_19_H_25_O), and 251.1785 (C_19_H_23_) by typical losses of 18 Da (H_2_O), 28 Da (CO), and 18 Da (H_2_O) in the MS^3^ spectrum. In addition, the loss of CO was apparent at *m*/*z* 315.1946 (*m*/*z* 315.1946 → 287.1998) and at *m*/*z* 297.1839 (*m*/*z* 297.1839 → 269.1890), and the loss of CH_2_O was apparent at *m*/*z* 315.1946 (*m*/*z* 315.1946 → 285.1840) in the MS^3^ spectra, suggesting that the methyleneoxy between C-7 and C-10 was also rearranged on the B-ring. The loss of H_2_O was also apparent at *m*/*z* 375.2150 (*m*/*z* 375.2150 → 357.2051), *m*/*z* 315.1946 (*m*/*z* 315.1946 → 297.1839), and *m*/*z* 287.1998 (*m*/*z* 287.1998 → 269.1890), proposing the cleavage of the hydroxyl group of 1α-acetoxy-14β-hydroxyl-7,20-epoxy-ent-kaur-16-en-15-one.

Compounds **3** and **7** are isomers of 1α-acetoxy-14β-hydroxyl-7,20-epoxy-ent-kaur-16-en-15-one and showed fragmentation behaviors similar to those of 1α-acetoxy-14β-hydroxyl-7,20-epoxy-ent-kaur-16-en-15-one (**13**). The typical losses of 18 Da (H_2_O), 60 Da (C_2_H_4_O_2_), 18 Da (H_2_O), 28 Da (CO), and 18 Da (H_2_O) were commonly observed, and most produced ions at *m*/*z* 357.2050 (C_22_H_29_O_4_), 315.1949 (C_20_H_27_O_3_), 297.1844 (C_20_H_25_O_2_), 269.1895 (C_19_H_25_O), and 251.1785 (C_19_H_23_).

Compounds **10** and **14** exhibited [M + H]^+^ at 317.2104 (C_20_H_29_O_3_), and both presented MS/MS characteristic ions at *m*/*z* 299.2000 (C_20_H_27_O_2_), 281.1896 (C_20_H_25_O), 271.2052 (C_19_H_27_O), and 253.1946 (C_19_H_25_). All were 58 Da smaller than the characteristic ions of 1α-acetoxy-14β-hydroxyl-7,20-epoxy-ent-kaur-16-en-15-one, suggesting that Compounds **10** and **14** represent 1α-acetoxy-14β-hydroxyl-7,20-epoxy-ent-kaur-16-en-15-one lacking the terminal acetoxy group (C_2_H_2_O_2_). Compounds **11** and **12** shared the common ion [M + H]^+^ at *m*/*z* 317.2101 (C_20_H_29_O_3_). The presence of ions at *m*/*z* 299.2000 and *m*/*z* 281.1899 indicates that both shared the same skeleton with Compound **10** and were isomers of Compound **10**. Notably, the same characteristic fragment ion at *m*/*z* 275.1637 (C_17_H_21_O_3_) indicates that the A-ring of Compounds **11** and **12** is prone to cleavage. Compounds **8** and **17** showed [M + H]^+^ precursor ions at *m*/*z* 315.1945 (C_20_H_27_O_3_) and common fragmentation behaviors, such as the ions at *m*/*z* 297.1842 (C_20_H_25_O_2_), 273.1484 (C_17_H_21_O_3_), and 269.1893 (C_19_H_25_O). All were 2 Da smaller than Compound **10** during their ESI-MS*^n^* fragmentations, suggesting that they represent Compound **10** with an additional double bond. Compound **9** gave rise to an [M + H]^+^ ion at *m*/*z* 331.1897 (C_20_H_27_O_4_) and fragment ions at *m*/*z* 313.1794 (C_20_H_25_O_3_), 289.1429 (C_17_H_21_O_4_), and 285.1844 (C_19_H_25_O_2_). All of them were 16 Da larger than Compound **8**, suggesting that it represents Compound **8** with a hydroxyl addition.

Compound **15** generated [M + H]^+^ ions at 357.2055 (C_22_H_29_O_4_) and fragment ions at *m*/*z* 297.1844 (C_20_H_25_O_2_), 255.1375 (C_17_H_19_O_2_), and 241.1218 (C_16_H_17_O_2_). The characteristic ions of Compound **15** were 18 Da (H_2_O) smaller than 1α-acetoxy-14β-hydroxyl-7,20-epoxy-ent-kaur-16-en-15-one, suggesting that it represents the reference Compound 1α-acetoxy-14β-hydroxyl-7,20-epoxy-ent-kaur-16-en-15-one lacking a hydroxyl group and double bond addition. Compound **16** had [M + H]^+^ at 359.2209 (C_22_H_31_O_4_), and it presented MS/MS characteristic ions at *m*/*z* 299.1996 (C_20_H_27_O_2_) and *m*/*z* 281.1897 (C_20_H_25_O). All were 16 Da smaller than the characteristic ions of 1α-acetoxy-14β-hydroxyl-7,20-epoxy-ent-kaur-16-en-15-one, suggesting that Compound **16** represents 1α-acetoxy-14β-hydroxyl-7,20-epoxy-ent-kaur-16-en-15-one lacking OH elements.

#### 2.2.2. Bridgehead-Substituted 7,20-Epoxy-Ent-Kaurane Diterpenoids

**Figure 6:**
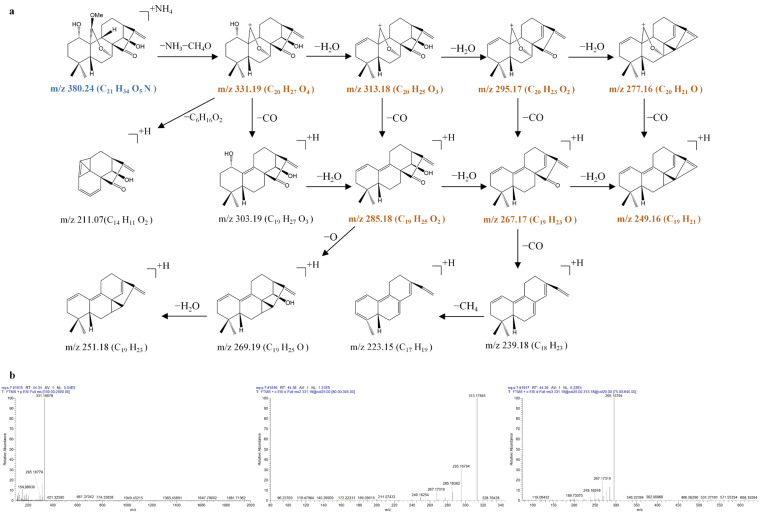
The fragmentation pathway of kamebacetal A recorded using a collision energy of 25 V (**a**) and its CID MS/MS spectrum (**b**).

**Figure 7:**
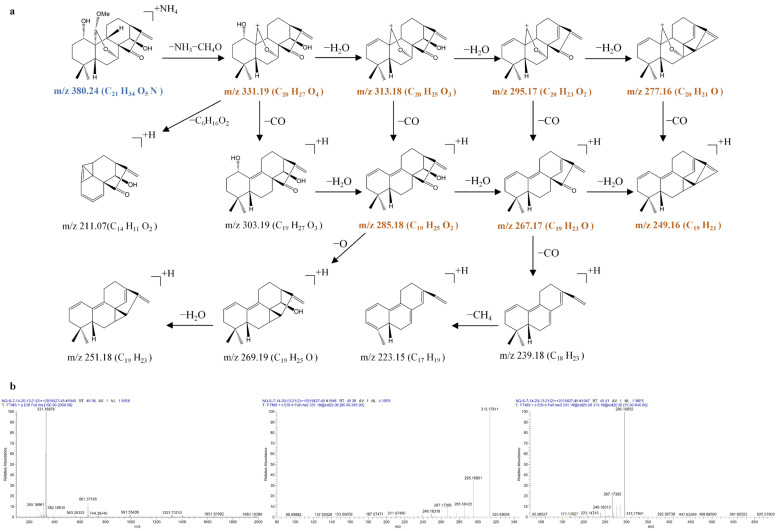
The fragmentation pathway of kamebacetal B recorded using a collision energy of 25 V (**a**) and its CID MS/MS spectrum (**b**).

**Figure 8:**
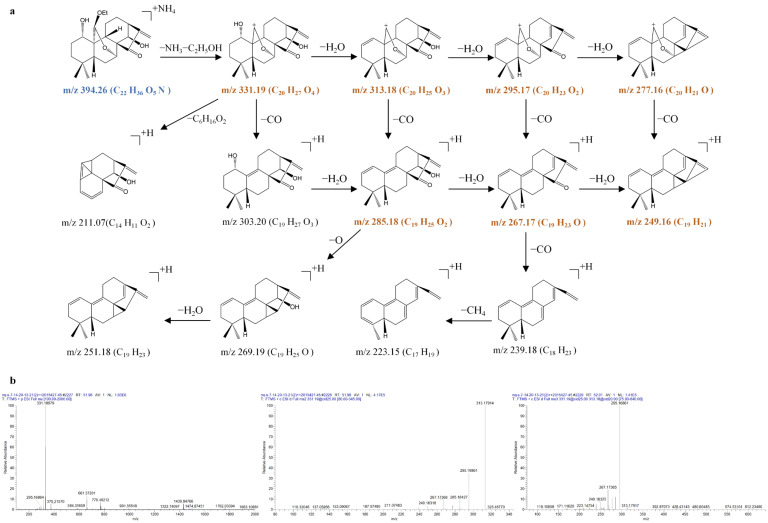
The fragmentation pathway of reniformin C recorded using a collision energy of 25 V (**a**) and its CID MS/MS spectrum (**b**).

The bridgehead-substituted 7,20-epoxy-ent-kaurane diterpenoids consisted of kamebacetal A (**21**), kamebacetal B (**22**), and reniformin C (**23**). The MS*^n^* mass spectrometry data and the fragmentation pathways of kamebacetal A (**21**), kamebacetal B (**22**), and reniformin C (**23**) are shown in Figure 6, Figure 7 and Figure 8.

Kamebacetal A (**21**) and kamebacetal B (**22**) are isomers, and both feature a methoxy group at the C-20 position. They generated the same [M + NH_4_ − NH_3_ − CH_4_O]^+^ precursor ions at 331.1895 (C_20_H_27_O_4_), very few [M + NH_4_]^+^ ions at *m*/*z* 380.2448 (C_21_H_34_O_5_N), and [2M + NH_4_]^+^ ions at *m*/*z* 742.4508 (C_42_H_64_O_10_N) in the MS^1^ spectra. The precursor ion at *m*/*z* 331.1895 could be fragmented to produce ions at *m*/*z* 313.1792 (C_20_H_25_O_3_), *m*/*z* 295.1686 (C_20_H_23_O_2_), *m*/*z* 267.1737 (C_19_H_23_O), and *m*/*z* 249.1632 (C_19_H_21_) through typical losses of 18 Da (H_2_O), 18 Da (H_2_O), 28 Da (CO), and 18 Da (H_2_O). Remarkably, the loss of CO was apparent at *m*/*z* 313.1792 (*m*/*z* 313.1792 → 285.1842), *m*/*z* 295.1686 (*m*/*z* 295.1686 → 267.1737), and *m*/*z* 277.1581 (*m*/*z* 277.1581 → 249.1632), suggesting that the methyleneoxy between C-7 and C-10 was rearranged on the B-ring. The loss of H_2_O was also apparent at *m*/*z* 331.1895 (*m*/*z* 331.1895 → 313.1792), *m*/*z* 313.1792 (*m*/*z* 313.1792 → 295.1686), and *m*/*z* 285.1842 (*m*/*z* 285.1842 → 267.1737), suggesting the cleavage of the hydroxyl groups of kamebacetal A and kamebacetal B.

Due to the existence of an ethoxy group at C-20, reniformin C (**23**) produced the [M + NH_4_ − NH_3_ − CH_3_CH_2_OH]^+^ precursor ions at 331.1898 (C_20_H_27_O_4_), very few [M + NH_4_]^+^ ions at *m*/*z* 394.2577 (C_22_H_36_O_5_N), and [2M + NH_4_]^+^ ions at *m*/*z* 770.4820 (C_44_H_68_O_10_N) in the MS^1^ spectra. Then, the precursor ion at *m*/*z* 331.1895 also further fragmented to produce ions at *m*/*z* 313.1792, 295.1686, 285.1842, 277.1581, 267.1737, and 249.1632, which are the same as those of kamebacetal A. Therefore, the [M + NH_4_ − NH_3_ − R]^+^ (R is the substituent group at the C-20 position) precursor ions at 331.1895 (C_20_H_27_O_4_), and the characteristic ions at *m*/*z* 313.1792, 295.1686, 285.1842, 277.1581, 267.1737, and 249.1632 were the most possible fragmentation pathways for bridgehead-substituted 7,20-epoxy-ent-kaurane diterpenoids.

Compounds **18**, **19**, and **20** generated the [M + NH_4_ − NH_3_ − H_2_O]^+^ precursor ions at 331.1896 (C_20_H_27_O_4_), [M + NH_4_]^+^ ions at *m*/*z* 366.2248 (C_20_H_32_O_5_N), and [2M + NH_4_]^+^ ions at *m*/*z* 714.4156 (C_40_H_60_O_10_N) in the MS^1^ spectra. Then, the precursor ion at *m*/*z* 331.1896 further fragmented to produce the characteristic ions at *m*/*z* 313.1792, 295.1688, 285.1844, 277.1582, 267.1738, and 249.1633, which are the same as those of kamebacetal A, indicating that the methoxy group at the C-20 position of kamebacetal A was replaced by the hydroxyl group. Based on previous reports [27], they were tentatively characterized as dimethyl kamebacetal A and its isomers.

#### 2.2.3. 7,20-Non-Epoxy-Kaurane Diterpenoids

**Figure 9:**
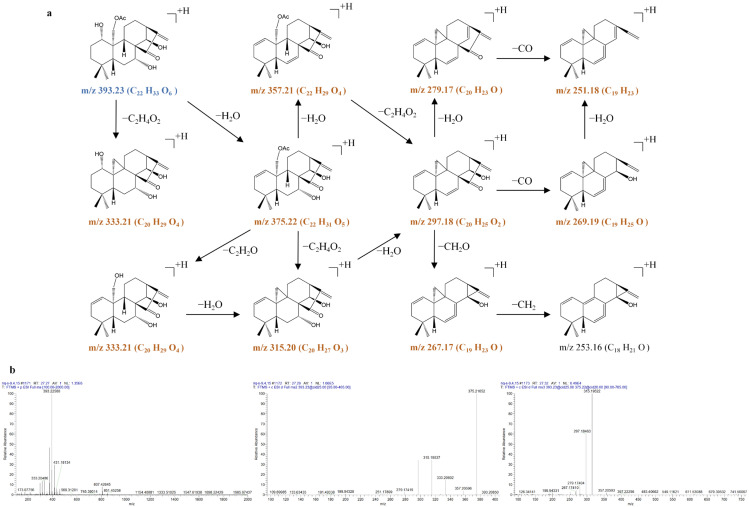
The fragmentation pathway of henryin recorded using a collision energy of 25 V (**a**) and its CID MS/MS spectrum (**b**).

**Figure 10:**
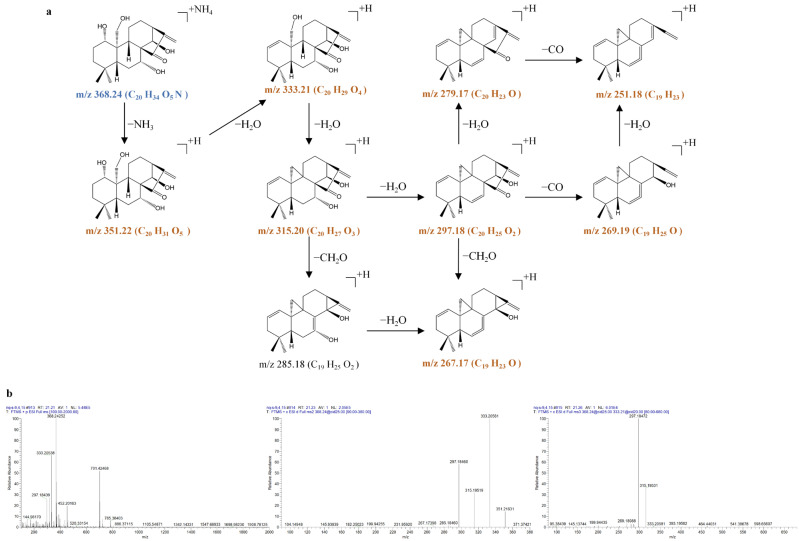
The fragmentation pathway of kamebakaurin recorded using a collision energy of 25 V (**a**) and its CID MS/MS spectrum (**b**).

**Figure 11:**
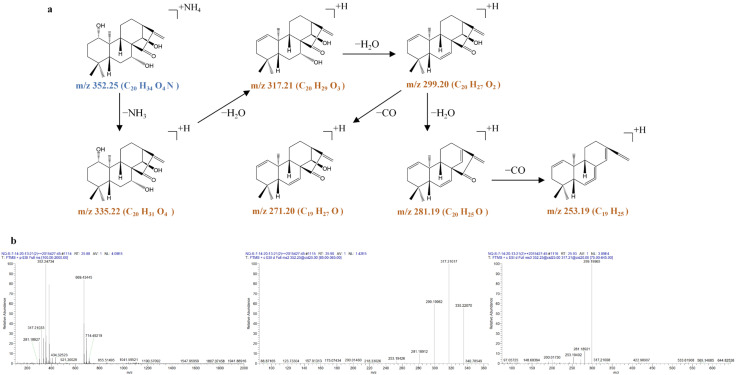
The fragmentation pathway of kamebanin recorded using a collision energy of 25 V (**a**) and its CID MS/MS spectrum (**b**).

**Figure 12:**
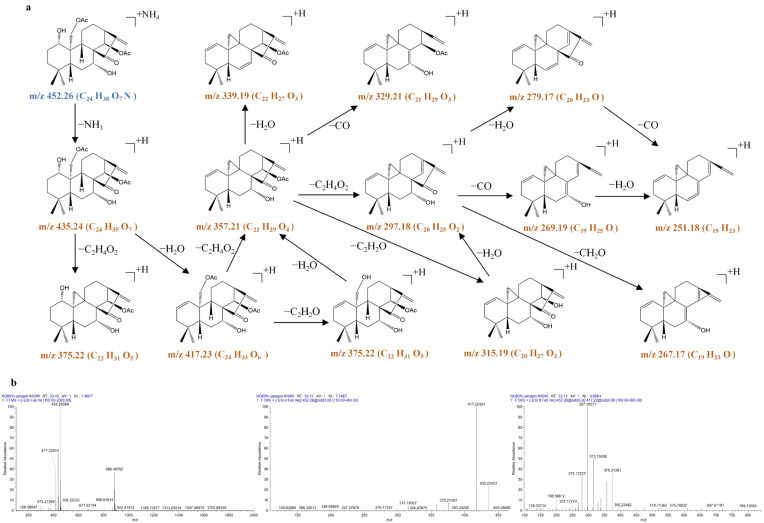
The fragmentation pathway of 1α,7α-dihydroxy-14β,20-diacetoxy-ent-kaur-16-en-15-one recorded using a collision energy of 25 V (**a**) and its CID MS/MS spectrum (**b**).

**Figure 13:**
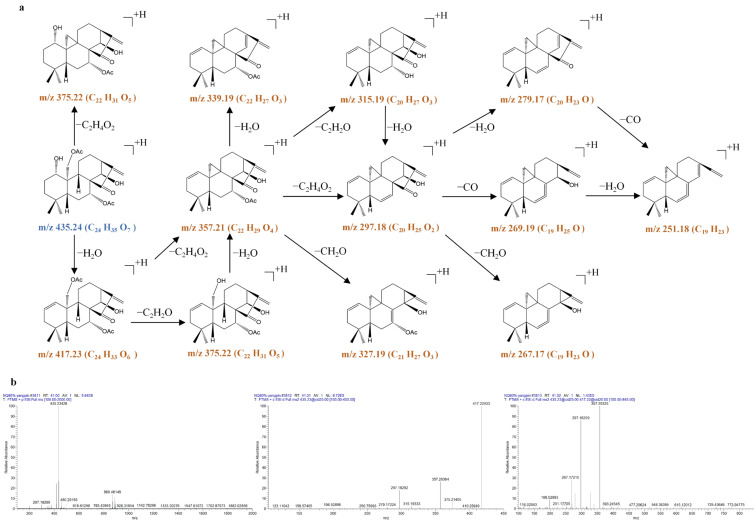
The fragmentation pathway of 1α,14β-dihydroxy-7α,20-diacetoxy-ent-kaur-16-en-15-one recorded using a collision energy of 25 V (**a**) and its CID MS/MS spectrum (**b**).

**Figure 14:**
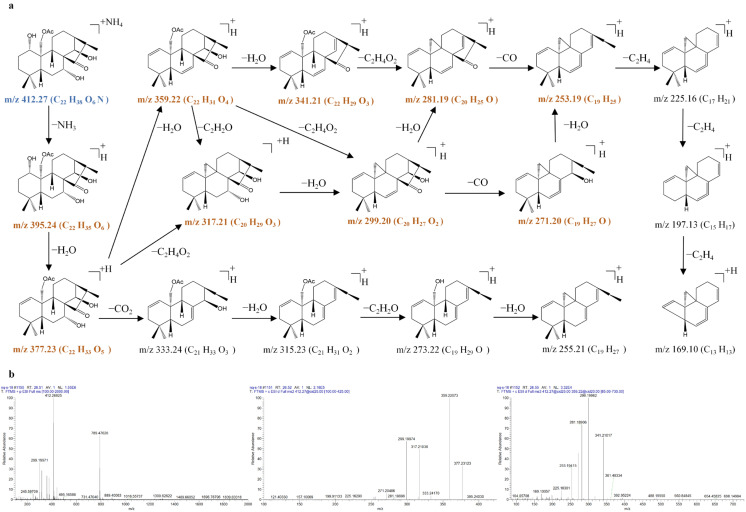
The fragmentation pathway of 1α,7α,14β-trihydroxy-20-acetoxy-ent-kaur-15-one recorded using a collision energy of 25 V (**a**) and its CID MS/MS spectrum (**b**).

Kamebakaurin (**44**), kamebanin (**52**), 1α,7α,14β-trihydroxy-20-acetoxy-ent-kaur-15-one (**54**), henryin (**57**), 1α,7α-dihydroxy-14β,20-diacetoxy-ent-kaur-16-en-15-one (**64**), and 1α,14β-dihydroxy-7α,20-diacetoxy-ent-kaur-16-en-15-one (**74**) are important 7,20-non-epoxy-kaurane diterpenoid components in IEH. The MS*^n^* mass spectrometry data and their fragmentation pathways are shown in Figure 9, Figure 10, Figure 11, Figure 12, Figure 13 and Figure 14.

Henryin (**57**) generated [M + H]^+^ precursor ions at 393.2258 (C_22_H_33_O_6_), [M + NH_4_]^+^ ions at *m*/*z* 410.2522 (C_22_H_36_O_6_N), and [2M + Na]^+^ ions at *m*/*z* 807.4265 (C_44_H_64_O_12_Na) in the MS^1^ spectra. Due to the existence of three hydroxyl groups at C-1, C-7, and C-14, an acetoxy group at C-20, and a carbonyl group at C-15 of henryin, the precursor ion at *m*/*z* 393.2258 further fragmented to produce the characteristic ions at *m*/*z* 375.2165 ([M + H − H_2_O]^+^), *m*/*z* 357.2060 ([M + H − 2H_2_O]^+^), *m*/*z* 333.2060 ([M + H − C_2_H_4_O_2_]^+^), *m*/*z* 315.1954 ([M + H − H_2_O − C_2_H_4_O_2_]^+^), *m*/*z* 297.1848 ([M + H − H_2_O − C_2_H_4_O_2_ − H_2_O]^+^), *m*/*z* 279.1742 ([M + H − H_2_O − C_2_H_4_O_2_ − 2H_2_O]^+^), *m*/*z* 269.1902 ([M + H − H_2_O − C_2_H_4_O_2_ − H_2_O − CO]^+^), *m*/*z* 267.1737 ([M + H − H_2_O − C_2_H_4_O_2_ − H_2_O − CH_2_O]^+^), and *m*/*z* 251.1790 ([M + H − H_2_O − C_2_H_4_O_2_ − 2H_2_O − CO]^+^).

Compounds **27**, **30**, **51**, **59**–**60**, and **66** are isomers of henryin and exhibited fragmentation behaviors similar to those of henryin (**57**). The typical losses of 18 Da (H_2_O), 60 Da (C_2_H_4_O_2_), and 28 Da (CO) were commonly observed, and most produced ions at *m*/*z* 375.2159 (C_22_H_31_O_5_), 357.2054 (C_22_H_29_O_4_), 315.1948 (C_20_H_27_O_3_), 297.1843 (C_20_H_25_O_2_), 279.1740 (C_20_H_23_O), 269.1893 (C_19_H_25_O), and 251.1789 (C_19_H_23_). Compound **89** showed an [M + NH_4_]^+^ precursor ion at 408.2370 (C_22_H_34_O_6_N) and then produced an [M + NH_4_ − NH_3_ − H_2_O]^+^ ion at 373.2001 (C_22_H_29_O_5_) through the loss of H_2_O. [M + NH_4_ − NH_3_ − H_2_O]^+^ could be further fragmented to produce the ions at *m*/*z* 355.1898 (C_22_H_27_O_4_), 331.1898 (C_20_H_27_O_4_), 313.1793 (C_20_H_25_O_3_), 295.1688 (C_20_H_23_O_2_), 277.1583 (C_20_H_21_O), 267.1739 (C_19_H_23_O), and 249.1634 (C_19_H_21_) via typical losses of 18 Da (H_2_O), 60 Da (C_2_H_4_O_2_), and 28 Da (CO) in the MS^3^ spectrum, suggesting that it represents the reference compound henryin lacking 2H, and it was tentatively identified as dehydro-henryin. Compounds **33** and **49** gave rise to the [M + NH_4_]^+^ ions at 426.2475 (C_22_H_36_O_7_N), and both presented MS/MS characteristic ions at *m*/*z* 391.2108 (C_22_H_31_O_6_), 373.2003 (C_22_H_29_O_5_), 331.1899 (C_20_H_27_O_4_), 313.1803 (C_20_H_25_O_3_), 295.1686 (C_20_H_23_O_2_), 285.1839 (C_19_H_25_O_2_), and 267.1743 (C_19_H_23_O). These characteristic ions were 16 Da (O) larger than henryin, suggesting that they represent henryin upon adding an OH group. Compound **82** had [M + NH_4_]^+^ ions at *m*/*z* 392.2422 (C_22_H_34_O_5_N) and an [M + H]^+^ ion at *m*/*z* 375.2156 (C_22_H_31_O_5_), which were 18 Da (H_2_O) smaller than henryin, and then, it produced the same characteristic ions of henryin at *m*/*z* 357.2053 (C_22_H_29_O_4_), 333.2054 (C_20_H_29_O_4_), 315.1949 (C_20_H_27_O_3_), 297.1843 (C_20_H_25_O_2_), 279.1737 (C_20_H_23_O), 269.11894 (C_19_H_25_O), and 251.1788 (C_19_H_23_), suggesting that it represents henryin lacking one hydroxyl group and double bond additions. Compound **92** gave rise to the [M + H]^+^ ion at *m*/*z* 785.4455 (C_44_H_65_O_12_), which was 392 Da (C_22_H_32_O_6_) larger than henryin (C_22_H_32_O_6_), and the MS*^n^* spectrum showed the characteristic fragment ions at *m*/*z* 767.4342 (C_44_H_63_O_11_, [M + H − H_2_O]^+^), 749.4239 (C_44_H_61_O_10_, [M + H − 2H_2_O]^+^), 707.4133 (C_42_H_59_O_9_, [M + H − H_2_O − C_2_H_4_O_2_]^+^), 689.4028 (C_42_H_57_O_8_, [M + H − H_2_O − C_2_H_4_O_2_ − H_2_O]^+^), 671.3923 (C_42_H_55_O_7_, [M + H − H_2_O − C_2_H_4_O_2_ − 2H_2_O]^+^), 661.4083 (C_41_H_57_O_7_, [M + H − H_2_O − C_2_H_4_O_2_ − H_2_O − CO]^+^), 643.3975 (C_41_H_55_O_6_, [M + H − H_2_O − C_2_H_4_O_2_ − H_2_O − CO − H_2_O]^+^), 393.2250 (C_22_H_33_O_6_, [M + H − C_22_H_32_O_6_]^+^), 375.2160 (C_22_H_31_O_5_, [M + H − C_22_H_32_O_6_ − H_2_O]^+^), 315.1948 (C_20_H_27_O_3_, [M + H − C_22_H_32_O_6_ − H_2_O − C_2_H_4_O_2_]^+^), 297.1840 (C_20_H_25_O_2_, [M + H − C_22_H_32_O_6_ − H_2_O − C_2_H_4_O_2_ − H_2_O]^+^), and 279.1733 (C_20_H_23_O, [M + H − C_22_H_32_O_6_ − H_2_O − C_2_H_4_O_2_ − 2H_2_O]^+^) via the successive loss of multiple 18 Da (H_2_O) and 60 Da (C_2_H_4_O_2_), suggesting that it represents the diploid of henryin; it was tentatively identified as dihenryin. Compound **29** produced the [M + NH_4_]^+^ ion at *m*/*z* 438.2841 (C_24_H_40_O_6_N), which was 28 Da (C_2_H_4_) larger than henryin, and the MS*^n^* spectrum showed the characteristic fragment ions at *m*/*z* 315.1945 (C_20_H_27_O_3_, [M + NH_4_ − NH_3_ − C_2_H_5_OH − C_2_H_4_O_2_]^+^), 297.1844 (C_20_H_25_O_2_, [M + NH_4_ − NH_3_ − C_2_H_5_OH − C_2_H_4_O_2_ − H_2_O]^+^), 279.1743 (C_20_H_23_O, [M + NH_4_ − NH_3_ − C_2_H_5_OH − C_2_H_4_O_2_ − 2H_2_O]^+^), and 251.17872 (C_19_H_23_O, [M + NH_4_ − NH_3_ − C_2_H_5_OH − C_2_H_4_O_2_ − 2H_2_O − CO]^+^), suggesting that the OH group at C-1 of henryin became the CH_3_CH_2_O group. Compound **87** produced the [M + H]^+^ ion at *m*/*z* 421.2570 (C_24_H_37_O_6_), which was also 28 Da (C_2_H_4_) larger than henryin. Notably, the MS*^n^* spectrum showed the characteristic fragment ions at *m*/*z* 403.2464 (C_24_H_35_O_5_, [M + H − H_2_O]^+^), 361.2367 (C_22_H_33_O_4_, [M + H − C_2_H_4_O_2_]^+^), 343.2255 (C_22_H_31_O_3_, [M + H − H_2_O − C_2_H_4_O_2_]^+^), 283.2046 (C_20_H_27_O, [M + H − H_2_O − C_2_H_4_O_2_ − C_2_H_4_O_2_]^+^), and 265.1947 (C_20_H_25_, [M + H − H_2_O − C_2_H_4_O_2_ − C_2_H_4_O_2_ − H_2_O]^+^) through the typical loss of the H_2_O and C_2_H_4_O_2_ groups, suggesting that the OH group at C-7 of henryin was replaced by a CH_3_CH_2_O group.

The only difference between henryin (**57**) and kamebakaurin (**44**) regarding chemical structure is that henryin has an OAc group at the C-20 position, while kamebakaurin has an OH group at the C-20 position. Therefore, kamebakaurin (**44**) produced an [M + NH_4_]^+^ ion at *m*/*z* 368.2425 (C_20_H_34_O_5_N), an [M + H]^+^ ion at *m*/*z* 351.2163 (C_20_H_31_O_5_), an [M + NH_4_ − NH_3_ − H_2_O]^+^ ion at *m*/*z* 333.2058 (C_20_H_29_O_4_), and an [M + NH_4_ − NH_3_ − 2H_2_O]^+^ ion at *m*/*z* 315.1952 (C_20_H_27_O_3_). All were 42 Da larger than henryin. The ion at *m*/*z* 333.2058 was further fragmented to produce prominent ions at *m*/*z* 315.1952 (C_20_H_27_O_3_), 297.1846 (C_20_H_25_O_2_), and 279.1740 (C_20_H_23_O) via successive losses of 18 Da (H_2_O) in the MS^3^ spectrum.

Compounds **24**, **32**, **35**–**37**, **40**, **43**, **50**, **53**, **62**, and **83** are isomers of kamebakaurin and showed fragmentation behaviors similar to those of kamebakaurin (**44**). The successive losses of 18 Da (H_2_O) were commonly observed, and most produced the characteristic ions at *m*/*z* 351.2161 (C_20_H_31_O_5_), 333.2056 (C_20_H_29_O_4_), 315.1951 (C_20_H_27_O_3_), 297.1846 (C_20_H_25_O_2_), and 279.1743 (C_20_H_23_O). Compounds **41**, **76**, and **81** have the same [M + NH_4_]^+^ ions at 366.2266 (C_20_H_32_O_5_N), with an [M + NH_4_ − NH_3_ − H_2_O]^+^ ion produced at 331.1897 (C_20_H_27_O_4_) through the loss of H_2_O. [M + NH_4_ − NH_3_ − H_2_O]^+^ could be further fragmented to produce ions at *m*/*z* 331.1897 (C_20_H_27_O_4_), 313.1792 (C_20_H_25_O_3_), and 295.1688 (C_20_H_23_O_2_) via successive losses of 18 Da (H_2_O) in the MS^3^ spectrum, suggesting that it represents the reference compound kamebakaurin lacking 2H, and it was tentatively identified as dehydro-kamebakaurin. Compound **48** had [M + NH_4_]^+^ ions at *m*/*z* 370.2580 (C_20_H_36_O_5_N) and presented MS*^n^* characteristic ions at *m*/*z* 335.2211 (C_20_H_31_O_4_), 317.2104 (C_20_H_29_O_3_), and 299.2000 (C_20_H_27_O_2_) through successive losses of 18 Da (H_2_O), suggesting that it represents kamebakaurin upon adding 2H, and it was tentatively identified as dihydro-kamebakaurin. Compounds **25** and **38** gave rise to an [M + NH_4_]^+^ ion at *m*/*z* 386.2529 (C_20_H_36_O_6_N) and the fragment ions at *m*/*z* 351.2159 (C_20_H_31_O_5_, [M + NH_4_ − NH_3_ − H_2_O]^+^), 333.2055 (C_20_H_29_O_4_, [M + NH_4_ − NH_3_ − 2H_2_O]^+^), 315.1951 (C_20_H_27_O_3_, [M + NH_4_ − NH_3_ − 3H_2_O]^+^), and 297.1838 (C_20_H_25_O_2_, [M + NH_4_ − NH_3_ − 4H_2_O]^+^). The characteristic ions of Compounds **25** and **38** were 18 Da (H_2_O) larger than kamebakaurin, suggesting that they represent the reference compound kamebakaurin with a double bond opening and hydroxyl addition. Compound **61** generated [M + NH_4_]^+^ ions at 398.2525 (C_21_H_36_O_6_N) and then produced the [M + NH_4_ − NH_3_]^+^ ion at 381.2261 (C_21_H_33_O_6_), [M + NH_4_ − NH_3_ − H_2_O]^+^ ion at 363.2157 (C_21_H_31_O_5_), and [M + NH_4_ − NH_3_ − 2H_2_O]^+^ ion at 345.2057 (C_21_H_29_O_4_). All were 30 Da (CH_2_O) larger than kamebakaurin. Furthermore, the characteristic fragmentations at *m*/*z* 331.1894 (C_20_H_27_O_4_) were from the ions at *m*/*z* 363.2157 (C_21_H_31_O_5_) through the loss of CH_4_O in the fragmentation pathways, indicating that Compound **61** represents the reference compound kamebakaurin upon adding a terminal methoxy group.

Compared with kamebakaurin, kamebanin (**52**) has no substituent at the C-20 position. Therefore, kamebanin (**52**) produced an [M + NH_4_]^+^ ion at *m*/*z* 352.2491 (C_20_H_34_O_4_N), an [M + H]^+^ ion at *m*/*z* 335.2223 (C_20_H_31_O_4_), an [M + NH_4_ − NH_3_ − H_2_O]^+^ ion at *m*/*z* 317.2118 (C_20_H_29_O_3_), and an [M + NH_4_ − NH_3_ − 2H_2_O]^+^ ion at *m*/*z* 299.2011 (C_20_H_27_O_2_). All were 16 Da smaller than kamebakaurin. The ion at *m*/*z* 317.2118 (C_20_H_29_O_3_) was further fragmented to produce prominent ions at *m*/*z* 299.2011 (C_20_H_27_O_2_) and 281.1906 (C_20_H_25_O) via successive losses of 18 Da (H_2_O) in the MS^3^ spectrum, suggesting the cleavage of hydroxyl groups. Significantly, both fragment ions at *m*/*z* 299.2011 (*m*/*z* 299.2011 → 271.2058) and at *m*/*z* 281.1906 (*m*/*z* 281.1906 → 253.1953) showed a small CO loss in the MS^3^ spectra, suggesting the cleavage of the carbonyl group.

Compounds **39**, **46**, **75**, and **84** are isomers of kamebanin and showed fragmentation behaviors similar to those of kamebanin (**52**). The typical losses of 18 Da (H_2_O) and 28 Da (CO) were commonly observed, and most produced characteristic ions at *m*/*z* 335.2193 (C_20_H_31_O_4_), 317.2092 (C_20_H_29_O_3_), 299.1987 (C_20_H_27_O_2_), 281.1877 (C_20_H_25_O), 271.2038 (C_19_H_27_O), and 253.1935 (C_20_H_25_O). Based on previous reports [11], Compound **39** was tentatively characterized as Wangzaozin A. Compound **28** has an [M + NH_4_]^+^ ion at *m*/*z* 532.3102 (C_26_H_46_O_10_N) and [M + H]^+^ ion at *m*/*z* 515.2832 (C_26_H_43_O_10_) through the typical loss of the NH_3_ group. The MS^2^ spectrum showed that the ion at *m*/*z* 515.2832 (C_26_H_43_O_10_) was cleaved to produce three prominent product ions at *m*/*z* 335.2213 (C_20_H_31_O_4_), 317.2100 (C_20_H_29_O_3_), and 299.1994 (C_20_H_27_O_2_) through typical losses of C_6_H_12_O_6_ and H_2_O, indicating that it represents kamebanin upon adding a terminal glucose monohydrate group (C_6_H_13_O_6_). Compounds **55** and **78** had [M + NH_4_]^+^ ions at *m*/*z* 354.2633 (C_20_H_36_O_4_N) and presented characteristic MS*^n^* ions at *m*/*z* 337.2369 (C_20_H_33_O_4_), 319.2263 (C_20_H_31_O_3_), 301.2157 (C_20_H_29_O_2_), and 283.2052 (C_20_H_27_O) through successive losses of 18 Da (H_2_O). All were 2 Da larger than kamebanin, suggesting that it represents kamebanin upon adding 2H and was tentatively identified as dihydro-kamebanin. Compound **80** gave rise to the [M + NH_4_]^+^ ions at 350.2318 (C_20_H_32_O_4_N) and then produced an [M + NH_4_−NH_3_]^+^ ion at 333.2054 (C_20_H_29_O_4_) through the loss of NH_3_. [M + NH_4_ − NH_3_]^+^ could be further fragmented to produce ions at *m*/*z* 315.1949 (C_20_H_27_O_3_) and 297.1849 (C_20_H_25_O_2_) via successive losses of 18 Da (H_2_O) in the MS^3^ spectrum, suggesting that it represents the reference compound kamebanin lacking 2H and was tentatively identified as dehydro-kamebanin. Compounds **31**, **47**, and **70** showed the same [M + H]^+^ ions at 333.2035 (C_20_H_29_O_4_) and then fragmented to produce ions at *m*/*z* 315.1934 (C_20_H_27_O_3_) and 297.1830 (C_20_H_25_O_2_) via the successive losses of 18 Da (H_2_O), similarly to Compound **80** in the MS^3^ spectrum, which indicates that these compounds were isomers of Compound **80**. Compound **71** generated [M + H]^+^ at 303.2312 (C_20_H_31_O_2_), and presented MS/MS characteristic ions at *m*/*z* 285.2208 (C_20_H_29_O) and *m*/*z* 267.2101 (C_20_H_27_). All were 32 Da smaller than the characteristic ions of kamebanin, suggesting that Compound **71** represents kamebanin lacking two OH elements. In addition, due to the fewer hydroxyl groups of Compound **71**, it was more likely to produce the fragment ions at *m*/*z* 239.1784 (C_18_H_23_), 225.1632 (C_17_H_21_), 211.1478 (C_16_H_19_), and 197.1320 (C_15_H_27_) through a series of cleavages and hydrogen rearrangements on the C-ring and D-ring, which result in successive losses of CH_2_.

1α,7α-dihydroxy-14β,20-diacetoxy-ent-kaur-16-en-15-one (**64**) produced the [M + NH_4_]^+^ precursor ion at *m*/*z* 452.2635 (C_24_H_38_O_7_N), very few [M + H]^+^ ions at *m*/*z* 435.2369 (C_24_H_35_O_7_), and the [2M + NH_4_]^+^ ion at *m*/*z* 886.4935 (C_48_H_72_O_14_N) in the MS^1^ spectra. The precursor ion at *m*/*z* 452.2635 (C_24_H_38_O_7_N) produced the [M + NH_4_ − NH_3_]^+^ ion at *m*/*z* 435.2369 (C_24_H_35_O_7_) through the typical loss of the NH_3_. Due to the existence of two hydroxyl groups at C-1 and C-7 and two acetoxy groups at C-14 and C-20, the [M + NH_4_ − NH_3_]^+^ ion at *m*/*z* 435.2369 was further fragmented to produce prominent ions at *m*/*z* 417.2269 (C_24_H_33_O_6_), *m*/*z* 375.2166 (C_22_H_31_O_5_), *m*/*z* 357.2052 (C_22_H_29_O_4_), *m*/*z* 315.1955 (C_20_H_27_O_3_), *m*/*z* 297.1848 (C_20_H_25_O_2_), and *m*/*z* 279.1742 (C_20_H_23_O) via typical losses of 18 Da (H_2_O), 42 Da (C_2_H_2_O), 18 Da (H_2_O), 42 Da (C_2_H_2_O), 18 Da (H_2_O), and 18 Da (H_2_O) in the MS^3^ spectrum.

Compounds **68**, **69**, and **77** are isomers of 1α,7α-dihydroxy-14β,20-diacetoxy-ent-kaur-16-en-15-one and showed fragmentation behaviors similar to those of 1α,7α-dihydroxy-14β,20-diacetoxy-ent-kaur-16-en-15-one (**64**). The typical losses of 17 Da (NH_3_), 18 Da (H_2_O), 42 Da (C_2_H_2_O), 18 Da (H_2_O), 42 Da (C_2_H_2_O), 18 Da (H_2_O), and 18 Da (H_2_O) were commonly observed, and most produced ions at *m*/*z* 435.2367 (C_24_H_35_O_7_), 417.2268 (C_24_H_33_O_6_),375.2157 (C_22_H_31_O_5_), 357.2053 (C_22_H_29_O_4_), 315.1947 (C_20_H_27_O_3_), 297.1842 (C_20_H_25_O_2_), and 279.1736 (C_20_H_23_O). Based on previous reports [9], they were tentatively characterized as 1α,14β-diacetoxy-7α,20-dihydroxy-ent-kaur-16-en-15-one, 1α,7α-diacetoxy-14β,20-dihydroxy-ent-kaur-16-en-15-one, and 1α,20-diacetoxy-7α,14β-dihydroxy-ent-kaur-16-en-15-one. Compound **85** exhibited [M + NH_4_]^+^ ions at 450.2474 (C_24_H_36_O_7_N) and then produced an [M + NH_4_ − NH_3_]^+^ ion at 433.2211 (C_24_H_33_O_7_) through the loss of NH_3_. [M + NH_4_ − NH_3_]^+^ could be further fragmented to produce ions at *m*/*z* 373.2002 (C_22_H_29_O_5_), 355.1894 (C_22_H_27_O_4_), 313.1794 (C_20_H_25_O_3_), 295.1689 (C_20_H_23_O_2_), and 277.1575 (C_20_H_21_O) via successive losses of 60 Da (C_2_H_4_O_2_), 18 Da (H_2_O), 42 Da (C_2_H_2_O), 18 Da (H_2_O), and 18 Da (H_2_O), suggesting that it represents the reference compound 1α,7α-dihydroxy-14β,20-diacetoxy-ent-kaur-16-en-15-one lacking 2H, and it was tentatively identified as dehydro-1α,7α-dihydroxy-14β,20-diacetoxy-ent-kaur-16-en-15-one. Moreover, the fragment ions at *m*/*z* 391.2106 (C_22_H_31_O_6_), 373.2000 (C_22_H_29_O_5_), 331.1897 (C_20_H_27_O_4_), and 313.1794 (C_20_H_25_O_3_) were produced from 433.2211 (C_24_H_33_O_7_) through the successive losses of 42 Da (C_2_H_2_O), 18 Da (H_2_O), 42 Da (C_2_H_2_O), and 18 Da (H_2_O), indicating that two acetoxy groups in Compound **85** were more prone to cleavage. Compound **58** generated [M + NH_4_]^+^ ions at *m*/*z* 454.2785 (C_24_H_40_O_7_N) and presented characteristic MS*^n^* ions at *m*/*z* 437.2524 (C_24_H_37_O_7_), 419.2414 (C_24_H_35_O_6_), *m*/*z* 377.2318 (C_22_H_33_O_5_), *m*/*z* 359.2211 (C_22_H_31_O_4_), *m*/*z* 317.2110 (C_20_H_29_O_3_), *m*/*z* 299.2001 (C_20_H_27_O_2_), and *m*/*z* 281.1896 (C_20_H_25_O) via typical losses of 17 Da (NH_3_), 18 Da (H_2_O), 42 Da (C_2_H_2_O), 18 Da (H_2_O), 42 Da (C_2_H_2_O), 18 Da (H_2_O), and 18 Da (H_2_O). All were 2 Da larger than 1α,7α-dihydroxy-14β,20-diacetoxy-ent-kaur-16-en-15-one, suggesting that it represents 1α,7α-dihydroxy-14β,20-diacetoxy-ent-kaur-16-en-15-one upon adding 2H, and it was tentatively identified as dihydro-1α,7α-dihydroxy-14β,20-diacetoxy-ent-kaur-16-en-15-one. Compound **73** exhibited the same [M + NH_4_]^+^ ions at *m*/*z* 454.2788 (C_24_H_40_O_7_N) and characteristic ions at *m*/*z* 437.2518 (C_24_H_37_O_7_), 419.2415 (C_24_H_35_O_6_), and 359.2209 (C_22_H_31_O_4_), indicating that it had similar fragmentation behaviors as Compound **58**. Therefore, this compound was identified as the isomer of Compound **58** in the extracted ion chromatogram. Compounds **45** and **56** gave rise to the [M + NH_4_]^+^ ion at *m*/*z* 470.2733 (C_24_H_40_O_8_N) and fragment ions at *m*/*z* 453.2468 (C_24_H_37_O_8_, [M + NH_4_ − NH_3_]^+^) and 393.2257 (C_22_H_33_O_6_, [M + NH_4_ − NH_3_ − C_2_H_4_O_2_]^+^). All were 18 Da (H_2_O) larger than 1α,7α-dihydroxy-14β,20-diacetoxy-ent-kaur-16-en-15-one, suggesting that they represent reference compound 1α,7α-dihydroxy-14β,20-diacetoxy-ent-kaur-16-en-15-one with a double bond opening and hydroxyl addition. However, Compound **45** also produced a relatively prominent characteristic ion at *m*/*z* 435.2366 (C_24_H_35_O_7_, [M + NH_4_ − NH_3_ − H_2_O]^+^), while Compound **56** generated relatively prominent ions at *m*/*z* 393.2262 (C_22_H_33_O_6_, [M + NH_4_ − NH_3_ − C_2_H_4_O_2_]^+^), 333.2053 (C_20_H_29_O_4_, [M + NH_4_ − NH_3_ − 2C_2_H_4_O_2_]^+^), and 315.1949 (C_20_H_27_O_3_, [M + NH_4_ − NH_3_ − 2C_2_H_4_O_2_ − H_2_O]^+^), indicating that the hydroxyl group of Compound **45** was more likely to cleave first, whereas the acetoxy groups of Compound **56** were more prone to cleavage. Compound **63** generated [M + NH_4_]^+^ ions at 484.2890 (C_25_H_42_O_8_N) and then produced [M + NH_4_ − NH_3_]^+^ ions at 467.2625 (C_25_H_39_O_8_) and [M + NH_4_ − NH_3_ − H_2_O]^+^ ions at 449.2519 (C_25_H_37_O_7_). All were 14 Da (CH_2_) larger than Compound **45**, indicating that Compound **63** represents Compound **45** upon adding a methyl group. Compound **86** gave rise to the [M + NH_4_]^+^ ion at *m*/*z* 496.2890 (C_26_H_42_O_8_N) and presented characteristic MS*^n^* ions at *m*/*z* 479.2627 (C_26_H_39_O_8_, [M + NH_4_ − NH_3_]^+^), *m*/*z* 461.2522 (C_26_H_37_O_7_, [M + NH_4_ − NH_3_ − H_2_O]^+^), *m*/*z* 419.2418 (C_24_H_35_O_6_, [M + NH_4_ − NH_3_ − H_2_O − C_2_H_2_O]^+^), *m*/*z* 401.2310 (C_24_H_33_O_5_, [M + NH_4_ − NH_3_ − H_2_O − C_2_H_2_O − H_2_O]^+^), *m*/*z* 377.2313 (C_22_H_33_O_5_, [M + NH_4_ − NH_3_ − H_2_O − 2C_2_H_2_O]^+^), *m*/*z* 359.2211 (C_22_H_31_O_4_, [M + NH_4_ − NH_3_ − H_2_O − 2C_2_H_2_O − H_2_O]^+^), *m*/*z* 341.2103 (C_22_H_29_O_3_, [M + NH_4_ − NH_3_ − H_2_O − 2C_2_H_2_O − H_2_O − H_2_O]^+^), *m*/*z* 317.2104 (C_20_H_29_O_3_, [M + NH_4_ − NH_3_ − H_2_O − 2C_2_H_2_O − H_2_O − C_2_H_2_O]^+^), *m*/*z* 299.1998 (C_20_H_27_O_2_, [M + NH_4_ − NH_3_ − H_2_O − 2C_2_H_2_O − H_2_O − C_2_H_2_O − H_2_O]^+^), and *m*/*z* 281.1893 (C_20_H_25_O, [M + NH_4_ − NH_3_ − H_2_O − 2C_2_H_2_O − H_2_O − C_2_H_2_O − 2H_2_O]^+^), suggesting that it represents 1α,7α-dihydroxy-14β,20-diacetoxy-ent-kaur-16-en-15-one upon adding a terminal OAc group (CH_3_CH_2_O). Compounds **79** and **88** showed the same [M + NH_4_]^+^ ions at *m*/*z* 496.2890 (C_26_H_42_O_8_N) in the positive mode and dominant ions at *m*/*z* 479.2623 (C_26_H_39_O_8_), *m*/*z* 461.2522 (C_26_H_37_O_7_), *m*/*z* 419.2415 (C_24_H_35_O_6_), *m*/*z* 401.2312 (C_24_H_33_O_5_), and *m*/*z* 359.2206 (C_22_H_31_O_4_). Therefore, they were tentatively identified as isomers of Compound **86**. Compound **94** gave rise to precursor ions at *m*/*z* 538.2992 (C_28_H_44_O_9_N, [M + NH_4_]^+^), which were 42 Da (C_2_H_2_O) larger than Compound **86**, and product ions at *m*/*z* 461.2521 (C_26_H_37_O_7_, [M + NH_4_ − NH_3_ − C_2_H_4_O_2_]^+^), *m*/*z* 419.2416 (C_24_H_35_O_6_, [M + NH_4_ − NH_3_ − C_2_H_4_O_2_ − C_2_H_2_O]^+^), *m*/*z* 401.2310 (C_24_H_33_O_5_, [M + NH_4_ − NH_3_ − C_2_H_4_O_2_ − C_2_H_2_O − H_2_O]^+^), *m*/*z* 359.2208 (C_22_H_31_O_4_, [M + NH_4_ − NH_3_ − C_2_H_4_O_2_ − 2C_2_H_2_O − H_2_O]^+^), *m*/*z* 341.2104 (C_22_H_29_O_3_, [M + NH_4_ − NH_3_ − C_2_H_4_O_2_ − 2C_2_H_2_O − H_2_O − H_2_O]^+^), *m*/*z* 299.1995 (C_20_H_27_O_2_, [M + NH_4_ − NH_3_ − C_2_H_4_O_2_ − 2C_2_H_2_O − H_2_O − C_2_H_2_O − H_2_O]^+^), and *m*/*z* 281.1893 (C_20_H_25_O, [M + NH_4_ − NH_3_ − C_2_H_4_O_2_ − 2C_2_H_2_O − H_2_O − C_2_H_2_O − 2H_2_O]^+^) through the typical loss of the C_2_H_4_O_2_, C_2_H_2_O and H_2_O groups, suggesting that one OH group on Compound **86** was converted to an OAc group (CH_3_CH_2_O).

1α,14β-dihydroxy-7α,20-diacetoxy-ent-kaur-16-en-15-one (**74**) and 1α,7α-dihydroxy-14β,20-diacetoxy-ent-kaur-16-en-15-one (**64**) are isomers. While 1α,14β-dihydroxy-7α,20-diacetoxy-ent-kaur-16-en-15-one (**74**) produced an [M + H]^+^ precursor ion at *m*/*z* 435.2373 (C_24_H_35_O_7_), very few [M + NH_4_]^+^ ions were observed at *m*/*z* 452.2635 (C_24_H_38_O_7_N), and a [2M + H]^+^ ion was observed at *m*/*z* 869.4676 (C_48_H_69_O_14_) in the MS^1^ spectra. Due to the existence of two hydroxyl groups at C-1 and C-14 and two acetoxy groups at C-7 and C-20, the [M + H]^+^ precursor ion at *m*/*z* 435.2373 was also further fragmented to produce prominent ions at *m*/*z* 417.2270 (C_24_H_33_O_6_), *m*/*z* 375.2164 (C_22_H_31_O_5_), *m*/*z* 357.2060 (C_22_H_29_O_4_), *m*/*z* 315.1954 (C_20_H_27_O_3_), *m*/*z* 297.1849 (C_20_H_25_O_2_), and *m*/*z* 279.1743 (C_20_H_23_O) via typical losses of 18 Da (H_2_O), 42 Da (C_2_H_2_O), 18 Da (H_2_O), 42 Da (C_2_H_2_O), 18 Da (H_2_O), and 18 Da (H_2_O).

Compound **72** showed the same [M + H]^+^ precursor ions at *m*/*z* 435.2365 (C_24_H_35_O_7_) and displayed common fragmentations, such as the ions at *m*/*z* 417.2261 (C_24_H_33_O_6_), *m*/*z* 357.2060 (C_22_H_29_O_4_), *m*/*z* 297.1849 (C_20_H_25_O_2_), and *m*/*z* 279.1738 (C_20_H_23_O), indicating that it was an isomer of 1α,14β-dihydroxy-7α,20-diacetoxy-ent-kaur-16-en-15-one. Compound **90** gave rise to the [M + H]^+^ ions at *m*/*z* 479.2621 (C_26_H_39_O_8_), which were 44 Da (C_2_H_4_O) larger than 1α,14β-dihydroxy-7α,20-diacetoxy-ent-kaur-16-en-15-one, and presented MS*^n^* characteristic ions at *m*/*z* 461.2519 (C_26_H_37_O_7_, [M + H − H_2_O]^+^), *m*/*z* 401.2308 (C_24_H_33_O_5_, [M + H − H_2_O − C_2_H_4_O_2_]^+^), *m*/*z* 341.2100 (C_22_H_29_O_3_, [M + H − H_2_O − 2C_2_H_4_O_2_]^+^), *m*/*z* 323.2003 (C_22_H_27_O_2_, [M + H − H_2_O − 2C_2_H_4_O_2_ − H_2_O]^+^), *m*/*z* 281.1892 (C_20_H_25_O, [M + H − H_2_O − 2C_2_H_4_O_2_ − H_2_O − C_2_H_2_O]^+^), and *m*/*z* 263.1795 (C_20_H_23_, [M + H − H_2_O − 2C_2_H_4_O_2_ − H_2_O − C_2_H_2_O − H_2_O]^+^), suggesting that it represents 1α,14β-dihydroxy-7α,20-diacetoxy-ent-kaur-16-en-15-one upon adding a terminal OAc group (CH_3_CH_2_O).

The full-scan mass spectrum of 1α,7α,14β-trihydroxy-20-acetoxy-ent-kaur-15-one (**54**) showed the [M + NH_4_]^+^ precursor ion at *m*/*z* 412.2683 (C_22_H_38_O_7_N), very few [M + H]^+^ ions at *m*/*z* 395.2417 (C_24_H_35_O_7_), and the [2M + H]^+^ ion at *m*/*z* 789.4762 (C_44_H_69_O_12_) in the MS^1^ spectra. The precursor ion at *m*/*z* 412.2683 (C_22_H_38_O_7_N) produced an [M + NH_4_ − NH_3_]^+^ ion at *m*/*z* 395.2425 (C_22_H_35_O_6_) through the typical loss of the NH_3_. Due to the existence of three hydroxyl groups at C-1, C-7, and C-14 and one acetoxy group at C-20, the [M + NH_4_ − NH_3_]^+^ ion at *m*/*z* 395.2425 was further fragmented to produce prominent ions at *m*/*z* 377.2313 (C_22_H_33_O_5_), *m*/*z* 359.2208 (C_22_H_31_O_4_), *m*/*z* 317.2103 (C_20_H_29_O_3_), *m*/*z* 299.1998 (C_20_H_27_O_2_), and *m*/*z* 281.1890 (C_20_H_25_O) via typical losses of 18 Da (H_2_O), 18 Da (H_2_O), 42 Da (C_2_H_2_O), 18 Da (H_2_O), and 18 Da (H_2_O).

Compound **67** is an isomer of 1α,7α,14β-trihydroxy-20-acetoxy-ent-kaur-15-one and showed fragmentation behaviors similar to those of 1α,7α,14β-trihydroxy-20-acetoxy-ent-kaur-15-one (**54**). The typical losses of 17 Da (NH_3_), 18 Da (H_2_O), 60 Da (C_2_H_4_O_2_), 18 Da (H_2_O), and 18 Da (H_2_O) were commonly observed, and this produced ions at *m*/*z* 395.2418 (C_22_H_35_O_6_), *m*/*z* 377.2297 (C_22_H_33_O_5_), *m*/*z* 317.2088 (C_20_H_29_O_3_), *m*/*z* 299.1983 (C_20_H_27_O_2_), and *m*/*z* 281.1871 (C_20_H_25_O). Compounds **26**, **34**, and **42** gave rise to the [M + NH_4_]^+^ ions at 428.2633 (C_22_H_38_O_7_N), and both presented MS/MS characteristic ions at *m*/*z* 411.2373 (C_22_H_35_O_7_), 393.2266 (C_22_H_33_O_6_), *m*/*z* 375.2161 (C_22_H_31_O_5_), *m*/*z* 333.2056 (C_20_H_29_O_4_), *m*/*z* 315.1949 (C_20_H_27_O_3_), and *m*/*z* 297.1842 (C_20_H_25_O_2_). These characteristic ions were 16 Da (O) larger than 1α,7α,14β-trihydroxy-20-acetoxy-ent-kaur-15-one, suggesting that they represent 1α,7α,14β-trihydroxy-20-acetoxy-ent-kaur-15-one upon adding an OH group. Compounds **65** and **93** had [M + NH_4_]^+^ ions at *m*/*z* 394.2559 (C_22_H_36_O_5_N) and [M + H]^+^ ions at *m*/*z* 377.2302 (C_22_H_33_O_5_), which were 18 Da (H_2_O) smaller than 1α,7α,14β-trihydroxy-20-acetoxy-ent-kaur-15-one; then, the same characteristic ions of 1α,7α,14β-trihydroxy-20-acetoxy-ent-kaur-15-one were produced at *m*/*z* 359.2192 (C_22_H_31_O_4_), 317.2090 (C_20_H_29_O_3_), and 299.1986 (C_20_H_27_O_2_), suggesting that they represent 1α,7α,14β-trihydroxy-20-acetoxy-ent-kaur-15-one lacking one hydroxyl group with double bond additions. Based on previous reports [3,12], they were tentatively characterized as leukamenin E and inflexanin A. Compound **91** gave rise to an [M + H]^+^ ion at *m*/*z* 771.4655 (C_44_H_67_O_11_), which was 18 Da (H_2_O) smaller than the [2M + H]^+^ ion of 1α,7α,14β-trihydroxy-20-acetoxy-ent-kaur-15-oneat *m*/*z* 789.4762 (C_44_H_69_O_12_), and the MS*^n^* spectrum showed characteristic fragment ions at *m*/*z* 753.4544 (C_44_H_65_O_10_, [M + H − H_2_O]^+^), 735.4453 (C_44_H_63_O_9_, [M + H − 2H_2_O]^+^), 717.4346 (C_44_H_61_O_8_, [M + H − H_2_O − 3H_2_O]^+^), 675.4242 (C_42_H_59_O_7_, [M + H − 3H_2_O − C_2_H_2_O]^+^), 657.4134 (C_42_H_57_O_6_, [M + H − 3H_2_O − C_2_H_4_O_2_]^+^), 639.4025 (C_42_H_55_O_5_, [M + H − 3H_2_O − C_2_H_4_O_2_ − H_2_O]^+^), 597.3923 (C_40_H_53_O_4_, [M + H − 3H_2_O − 2C_2_H_4_O_2_]^+^), 579.3824 (C_40_H_51_O_3_, [M + H − 3H_2_O − 2C_2_H_4_O_2_ − H_2_O]^+^), 561.3722 (C_40_H_49_O_2_, [M + H − 3H_2_O − 2C_2_H_4_O_2_ − 2H_2_O]^+^), and 393.2262 (C_22_H_33_O_6_, [M + H − C_22_H_34_O_5_]^+^) via the successive loss of multiple 18 Da (H_2_O), 60 Da (C_2_H_4_O_2_), and 378 Da (C_22_H_34_O_5_), suggesting that it represents the diploid of 1α,7α,14β-trihydroxy-20-acetoxy-ent-kaur-15-one lacking H_2_O.

## 3. Materials and Methods

### 3.1. Chemicals and Reagents

The standards—including the bridgehead-unsubstituted 7,20-epoxy-ent-kaurane diterpenoids of oridonin (**2**), lasiokaurin (**5**), and 1α-acetoxy-14β-hydroxyl-7,20epoxy-ent-kaur-16-en-15-one (**13**); bridgehead-substituted 7,20-epoxy-ent-kaurane diterpenoids of kamebacetal A (**21**), kamebacetal B (**22**), and reniformin C (**23**); and 7,20-non-epoxy-kaurane diterpenoids of kamebakaurin (**44**), kamebanin (**52**), 1α,7α,14β-trihydroxy-20-acetoxy-ent-kaur-15-one (**54**), henryin (**57**), 1α,7α-dihydroxy-14β,20-diacetoxy-ent-kaur-16-en-15-one (**64**), and 1α,14β-dihydroxy-7α,20-diacetoxy-ent-kaur-16-en-15-one (**74**)—were isolated from the IEH in our laboratory. Their structures were unequivocally elucidated via spectroscopic methods (IR, MS, ^1^H NMR, and ^13^C NMR). The purity of the standards was determined to be higher than 98% by normalizing the peak area using HPLC-DAD (diode array detector). Stock solutions (0.1 mg/mL) of the 12 standards were prepared individually in methanol. Acetonitrile (ACN) and formic acid (both HPLC grade) were purchased from Fisher Scientific (Fairlawn, NJ, USA). Methanol (HPLC grade) was purchased from Merck (Darmstadt, Germany). Deionized water was prepared by passing distilled water through a Milli-Q water purification system (Millipore, Milford, MA, USA).

### 3.2. Plant Material

Isodonis Excisoidis Herba were collected from Luanchuan, Henan, China (GPS coordinates 33°46′19.6″ N 111°44′59.2″ E) in July 2022 and authenticated by one of the authors, Prof. Liping Dai (Department of Pharmacognosy, Henan University of Chinese Medicine), according to morphological characteristics. A voucher specimen (voucher number 20220721) was deposited at the Pharmacognosy Lab of the Pharmacy College, Henan University of Chinese Medicine, Henan, China.

### 3.3. Sample Preparation

Isodonis Excisoidis Herba samples were pulverized into powder (1.0 g, 60 mesh), weighed, soaked in 10 mL of 80% ethanol at room temperature, and ultrasonically extracted for 1 h. The extracts were filtered through filter paper, and 5 mL of the successive filtrate was evaporated to dryness. The dried residue was redissolved in 1ml of methanol in a volumetric flask. All samples were filtered through a 0.22 μm membrane and directly analyzed using UHPLC-LTQ-Orbitrap-MS (Thermo Fisher Scientific, Bremen, Germany).

### 3.4. UHPLC Separation Conditions

The chromatographic analysis was performed on a Dionex UltiMate 3000 UHPLC system (Thermo Scientific, Germering, Bavaria, Germany) equipped with a binary pump, an online degasser, a thermostatted autosampler, a thermostatically controlled column compartment, and a diode array detector (DAD). Chromatographic separation was performed on a reverse-phase ACQUITY UPLC BEH-C18 column (2.1 × 100 mm, 1.7 μm, Waters) and maintained at 30 °C. The mobile phases consisted of deionized water containing 0.1% formic acid (A) and acetonitrile (B), and the elution gradient was set as follows: 0.0–10.0 min, 10.0% B; 10.0–15.0 min, 10.0–20.0% B; 15.0–20.0 min, 20.0% B; 20.0–36.0 min, 20.0–21.0% B; 36.0–37.0 min, 21.0–29.0% B; 37.0–43.0 min, 29% B; 43.0–48.0 min, 29.0–35.0% B; 48.0–52.0 min, 35.0–40.0% B; 52.0–62.0 min, 40.0% B; 62.0–72.0 min, 40.0–55.0% B; 72.0–75.0 min, 55.0–10.0% B; 75.0–80.0 min, 10% B. The flow rate was set at 0.3 mL/min, and the injection volume was 2 μL.

### 3.5. Mass Spectrometry and Data Processing

For the LC-ESI-MS*^n^* experiments, a Thermo Fisher LTQ-Orbitrap XL Hybrid mass spectrometer (Thermo Fisher Scientific, Bremen, Germany) equipped with an electrospray ionization (ESI) source was connected to the UHPLC instrument. The ESI source parameters were set as follows: ion spray voltage, 4.2 kV; capillary temperature, 300 °C; capillary voltage, 36 V; tube lens voltage, 110 V; and sheath (N_2_) and auxiliary gas (He) flow rates of 40 and 10 arbitrary units, respectively. The Orbitrap mass analyzer was operated in the positive ion mode, with a mass range of 100 to 2000. Tuning methods were developed using a multi-objective optimization experiment, similarly to the technique described for ESI-MS. Accurate masses were calibrated according to the manufacturer’s guidelines using a standard mixture of caffeine, MRFA, and Ultramark 1621 in the positive ion mode.

The Fourier transform resolutions were set at 30,000 (full width at half maximum, as defined at *m*/*z* 400) for MS and MS*^n^* (*n* = 3). The MS and MS*^n^* data were recorded in profile and centroid formats, respectively. The average acquisition time required to finish a scan cycle (containing four scan events) was 3.6 s. The most intense ions detected in the full-scan spectrum were selected for the data-dependent scan. The normalized collision energy for CID was adjusted to 25% of the maximum in MS^2^ and 20% of the maximum in MS^3^, the isolation width of the precursor ions was *m*/*z* 2.0, and the default values were used for other CID parameters. To simultaneously obtain fragment information for co-eluting minor peaks, dynamic exclusion was enabled using the following optimal parameters: repeat count, 2; repeat duration, 10 s; exclusion duration, 10 s.

The data were recorded and processed using the Xcalibur 3.0 software (Thermo Fisher Scientific, Waltham, MA, USA) and Mass Frontier 7.0 software (Thermo Fisher Scientific, Waltham, MA, USA). Considering the possible elemental compositions of the potential components present in the IEH samples, the elements in use (C: 0–60; H: 0–100; O: 0–25; N: 0–2), ring double-bond equivalent (RDB equivalent value: −1.0–100.0), and mass tolerance (<10 ppm) were set to reduce the number of options used to determine the elemental compositions of both the precursor and product ions.

## 4. Conclusions

An efficient and sensitive method employing UHPLC-LTQ-Orbitrap-MS was developed for the qualitative profiling of the chemical constituents of IEH in the positive ion mode. By combining a wide range of information—including the exact mass from LTQ-Orbitrap-MS, fragmentation patterns, and the retention time on UHPLC—and comparing the results with reference substances and the literature, a total of 94 compounds, including 17 bridgehead-unsubstituted 7,20-epoxy-ent-kaurane diterpenoids, 6 bridgehead-substituted 7,20-epoxy-ent-kaurane diterpenoids, and 71 7,20-non-epoxy-kaurane diterpenoids, 48 of which are potentially new ent-kaurane diterpenoids, were detected from IEH. This is the first report on ent-kaurane diterpenoids in IEH using UHPLC-LTQ-Orbitrap-MS. The results not only provide abundant information for the identification and better understanding of the chemical constituents of IEH but also offer valuable insights for target-guided preparation, bioactivity-guided research, quality control, pharmacokinetic investigations, and metabolic studies of this Chinese medicine. This study also suggested that UHPLC-LTQ-Orbitrap-MS would be a powerful and reliable analytical tool for the characterization of chemical profiles in complex chemical systems, such as herbal medicines and TCM preparations. However, it must be emphasized that this technique has inherent limitations in resolving stereochemistry and regioisomerism. Consequently, the proposed structures, especially those of potentially new ent-kaurane diterpenoids, require definitive structural confirmation through isolation and comprehensive NMR analysis.

## Figures and Tables

**Table 1:** MS*^n^* data and proposed fragmentation pathways of the 12 reference compounds.

Type	Standard	Selection	Precursor Ions	Molecular Formula	Error (ppm)	MS^*n*^	Fragment Ions	Elemental Comp.	Error (ppm)	Pathways
Bridgehead-unsubstituted 7,20-epoxy-ent-kaurane diterpenoids	Oridonin (**2**)	[M + H]^+^	365.19547	C_20_H_29_O_6_	−1.082	MS^2^[365.20]	347.18503	C_20_H_27_O_5_	−0.779	[M + H − H_2_O]^+^
						MS^3^[347.19]	329.17453	C_20_H_25_O_4_	−0.625	[M + H − 2H_2_O]^+^
							311.16391	C_20_H_23_O_3_	−0.839	[M + H − 3H_2_O]^+^
							301.17957	C_19_H_25_O_3_	−0.834	[M + H − 2H_2_O − CO]^+^
							299.16394	C_19_H_23_O_3_	−0.772	[M + H − 2H_2_O − CH_2_O]^+^
							293.15350	C_20_H_21_O_2_	−0.363	[M + H − 4H_2_O]^+^
							283.16901	C_19_H_23_O_2_	−0.870	[M + H − 2H_2_O − CO − H_2_O]^+^
							281.15338	C_19_H_21_O_2_	−0.805	[M + H − 2H_2_O − CH_2_O − H_2_O]^+^
							273.18451	C_18_H_25_O_2_	−1.452	[M + H − 2H_2_O − 2CO]^+^
							265.15848	C_19_H_21_O	−0.799	[M + H − 3H_2_O − CO − H_2_O]^+^
							255.17409	C_18_H_23_O	−0.987	[M + H − 2H_2_O − 2CO − H_2_O]^+^
							247.14769	C_19_H_19_	−1.769	[M + H − 3H_2_O − CO − H_2_O − H_2_O]^+^
							237.16335	C_18_H_21_	−1.801	[M + H − 3H_2_O − CO − H_2_O − CO]^+^
	Lasiokaurin (**5**)	[M + H]^+^	407.20526	C_22_H_31_O_7_	−2.873	MS^2^[407.21]	389.19492	C_22_H_29_O_6_	−2.441	[M + H − H_2_O]^+^
							347.18422	C_20_H_27_O_5_	−3.112	[M + H − C_2_H_4_O_2_]^+^
						MS^3^[389.19]	371.18428	C_22_H_27_O_5_	−2.748	[M + H − 2H_2_O]^+^
							361.19980	C_21_H_29_O_5_	−3.185	[M + H − H_2_O − CO]^+^
							343.18951	C_21_H_27_O_4_	−2.552	[M + H − H_2_O − CO − H_2_O]^+^
							329.17390	C_20_H_25_O_4_	−2.539	[M + H − H_2_O − C_2_H_4_O_2_]^+^
							311.16335	C_20_H_23_O_3_	−2.639	[M + H − H_2_O − C_2_H_4_O_2_ − H_2_O]^+^
							301.17896	C_19_H_25_O_3_	−2.859	[M + H − H_2_O − C_2_H_4_O_2_ − CO]^+^
							299.16343	C_19_H_23_O_3_	−2.477	[M + H − H_2_O − C_2_H_4_O_2_ − CH_2_O]^+^
							293.15269	C_20_H_21_O_2_	−3.126	[M + H − H_2_O − C_2_H_4_O_2_ − 2H_2_O]^+^
							283.16840	C_19_H_23_O_2_	−3.025	[M + H − H_2_O − C_2_H_4_O_2_ − H_2_O − CO]^+^
							281.15285	C_19_H_21_O_2_	−2.690	[M + H − H_2_O − C_2_H_4_O_2_ − CH_2_O − H_2_O]^+^
							265.15780	C_19_H_21_O	−3.363	[M + H − H_2_O − C_2_H_4_O_2_ − H_2_O − CO − H_2_O]^+^
							247.14750	C_19_H_19_	−2.538	[M + H − H_2_O − C_2_H_4_O_2_ − H_2_O − CO − 2H_2_O]^+^
							237.16311	C_18_H_21_	−2.813	[M + H − H_2_O − C_2_H_4_O_2_ − H_2_O − CO − H_2_O − CO]^+^
							223.14750	C_17_H_19_	−2.811	[M + H − H_2_O − C_2_H_4_O_2_ − H_2_O − CO − 2H_2_O − C_2_]^+^
	1α-acetoxy-14β-hydroxyl-7,20-epoxy-ent-kaur-16-en-15-one (**13**)	[M + H]^+^	375.21570	C_22_H_31_O_5_	−2.400	MS^2^[375.22]	357.20512	C_22_H_29_O_4_	−2.564	[M + H − H_2_O]^+^
							329.17385	C_20_H_25_O_4_	−2.691	[M + H − H_2_O − C_2_H_4_]^+^
							315.19457	C_20_H_27_O_3_	−2.859	[M + H − C_2_H_4_O_2_]^+^
							285.18404	C_19_H_25_O_2_	−3.038	[M + H − C_2_H_4_O_2_ − CH_2_O]^+^
							267.17347	C_19_H_23_O	−3.263	[M + H − C_2_H_4_O_2_ − CH_2_O − H_2_O]^+^
						MS^3^[315.19]	297.18391	C_20_H_25_O_2_	−3.353	[M + H − C_2_H_4_O_2_ − H_2_O]^+^
							287.19979	C_19_H_27_O_2_	−2.669	[M + H − C_2_H_4_O_2_ − CO]^+^
							279.17335	C_20_H_23_O	−3.553	[M + H − C_2_H_4_O_2_ − 2H_2_O]^+^
							269.18905	C_19_H_25_O	−3.499	[M + H − C_2_H_4_O_2_ − H_2_O − CO]^+^
							255.13706	C_17_H_19_O_2_	−3.513	[M + H − C_2_H_4_O_2_ − H_2_O − C_3_H_6_]^+^
							251.17857	C_19_H_23_	−3.413	[M + H − C_2_H_4_O_2_ − H_2_O − CO − H_2_O]^+^
							241.12133	C_16_H_17_O_2_	−4.049	[M + H − C_2_H_4_O_2_ − H_2_O − C_4_H_8_]^+^
							223.14756	C_17_H_19_	−2.542	[M + H − C_2_H_4_O_2_ − H_2_O − CO − H_2_O − C_2_H_4_]^+^
							195.11619	C_15_H_15_	−3.265	[M + H − C_2_H_4_O_2_ − H_2_O − CO − H_2_O − 2C_2_H_4_]^+^
							149.13191	C_11_H_17_	−3.803	[M + H − C_2_H_4_O_2_ − H_2_O − CO − H_2_O − C_8_H_6_]^+^
							147.11626	C_11_H_15_	−3.854	[M + H − C_2_H_4_O_2_ − H_2_O − CO − H_2_O − C_8_H_8_]^+^
							121.10061	C_9_H_13_	−4.682	[M + H − C_2_H_4_O_2_ − H_2_O − CO − H_2_O − C_8_H_6_ − C_2_H_4_]^+^
Bridgehead-substituted 7,20-epoxy-ent-kaurane diterpenoids	Kamebacetal A (**21**)	[M + H − CH_4_O]^+^	331.18945	C_20_H_27_O_4_	−2.826	MS^2^[331.19]	313.17917	C_20_H_25_O_3_	−2.079	[M + H − CH_4_O − H_2_O]^+^
		[2M + NH_4_]^+^	742.44983	C_42_H_64_O_10_N	−3.561		303.19489	C_19_H_27_O_3_	−1.917	[M + H − CH_4_O − CO]^+^
		[M + NH_4_]^+^	380.24094	C_21_H_34_O_5_N	−5.811		287.16333	C_18_H_23_O_3_	−2.929	[M + H − CH_4_O − H_2_O − C_2_H_2_]^+^
							283.16837	C_19_H_23_O_2_	−3.131	[M + H − CH_4_O − H_2_O − CH_2_O]^+^
							211.07478	C_14_H_11_O_2_	−2.730	[M + H − CH_4_O − C_6_H_16_O_2_]^+^
						MS^3^[313.18]	295.16855	C_20_H_23_O_2_	−2.394	[M + H − CH_4_O − 2H_2_O]^+^
							285.18420	C_19_H_25_O_2_	−2.478	[M + H − CH_4_O − CO − H_2_O]^+^
							277.15793	C_20_H_21_O	−2.749	[M + H − CH_4_O − 3H_2_O]^+^
							269.18909	C_19_H_25_O	−3.351	[M + H − CH_4_O − CO − H_2_O − O]^+^
							267.17355	C_19_H_23_O	−2.964	[M + H − CH_4_O − CO − 2H_2_O]^+^
							259.14761	C_20_H_19_	−1.996	[M + H − CH_4_O − 4H_2_O]^+^
							257.11627	C_16_H_17_O_3_	−3.698	[M + H − CH_4_O − H_2_O − C_4_H_8_]^+^
							253.15836	C_18_H_21_O	−1.311	[M + H − CH_4_O − CO − 2H_2_O − CH_2_]^+^
							251.17877	C_19_H_23_	−2.617	[M + H − CH_4_O − CO − H_2_O − O − H_2_O]^+^
							249.16306	C_19_H_21_	−2.879	[M + H − CH_4_O − 3H_2_O − CO]^+^
							239.17873	C_18_H_23_	−2.915	[M + H − CH_4_O − 2CO − 2H_2_O]^+^
							223.14755	C_17_H_19_	−2.586	[M + H − CH_4_O − 2CO − 2H_2_O − CH_4_]^+^
	Kamebacetal B (**22**)	[M + H − CH_4_O]^+^	331.18976	C_20_H_27_O_4_	−1.890	MS^2^[331.19]	313.17911	C_20_H_25_O_3_	−2.271	[M + H − CH_4_O − H_2_O]^+^
		[2M + NH_4_]^+^	742.45300	C_42_H_64_O_10_N	0.709		303.19470	C_19_H_27_O_3_	−2.544	[M + H − CH_4_O − CO]^+^
		[M + NH_4_]^+^	380.24214	C_21_H_34_O_5_N	−2.655		287.16345	C_18_H_23_O_3_	−2.511	[M + H − CH_4_O − H_2_O − C_2_H_2_]^+^
							283.16861	C_19_H_23_O_2_	−2.283	[M + H − CH_4_O − H_2_O − CH_2_O]^+^
							211.07480	C_14_H_11_O_2_	−2.635	[M + H − CH_4_O − C_6_H_16_O_2_]^+^
						MS^3^[313.18]	295.16855	C_20_H_23_O_2_	−2.394	[M + H − CH_4_O − 2H_2_O]^+^
							285.18414	C_19_H_25_O_2_	−2.688	[M + H − CH_4_O − CO − H_2_O]^+^
							277.15796	C_20_H_21_O	−2.640	[M + H − CH_4_O − 3H_2_O]^+^
							269.18924	C_19_H_25_O	−2.793	[M + H − CH_4_O − CO − H_2_O − O]^+^
							267.17365	C_19_H_23_O	−2.590	[M + H − CH_4_O − CO − 2H_2_O]^+^
							259.14758	C_20_H_19_	−2.111	[M + H − CH_4_O − 4H_2_O]^+^
							257.11649	C_16_H_17_O_3_	−2.843	[M + H − CH_4_O − H_2_O − C_4_H_8_]^+^
							253.15797	C_18_H_21_O	−2.851	[M + H − CH_4_O − CO − 2H_2_O − CH_2_]^+^
							251.17863	C_19_H_23_	−3.174	[M + H − CH_4_O − CO − H_2_O − O − H_2_O]^+^
							249.16313	C_19_H_21_	−2.598	[M + H − CH_4_O − 3H_2_O − CO]^+^
							239.17865	C_18_H_23_	−3.250	[M + H − CH_4_O − 2CO − 2H_2_O]^+^
							223.14743	C_17_H_19_	−3.124	[M + H − CH_4_O − 2CO − 2H_2_O − CH_4_]^+^
	Reniformin C (**23**)	[M + H − C_2_H_5_OH]^+^	331.18979	C_20_H_27_O_4_	−1.799	MS^2^[331.19]	313.17914	C_20_H_25_O_3_	−2.175	[M + H − C_2_H_5_OH − H_2_O]^+^
		[2M + NH_4_]^+^	770.48199	C_44_H_68_O_10_N	−2.315		303.19507	C_19_H_27_O_3_	−1.323	[M + H − C_2_H_5_OH − CO]^+^
		[M + NH_4_]^+^	394.25772	C_22_H_36_O_5_N	−2.739		287.16364	C_18_H_23_O_3_	−1.849	[M + H − C_2_H_5_OH − H_2_O − C_2_H_2_]^+^
							283.16855	C_19_H_23_O_2_	−2.495	[M + H − C_2_H_5_OH − H_2_O − CH_2_O]^+^
							211.07483	C_14_H_11_O_2_	−2.493	[M + H − C_2_H_5_OH − C_6_H_16_O_2_]^+^
						MS^3^[313.18]	295.16861	C_20_H_23_O_2_	−2.190	[M + H − C_2_H_5_OH − 2H_2_O]^+^
							285.18417	C_19_H_25_O_2_	−2.583	[M + H − C_2_H_5_OH − CO − H_2_O]^+^
							277.15799	C_20_H_21_O	−2.532	[M + H − C_2_H_5_OH − 3H_2_O]^+^
							269.18939	C_19_H_25_O	−2.236	[M + H − C_2_H_5_OH − CO − H_2_O − O]^+^
							267.17365	C_19_H_23_O	−2.590	[M + H − C_2_H_5_OH − CO − 2H_2_O]^+^
							259.14746	C_20_H_19_	−2.574	[M + H − C_2_H_5_OH − 4H_2_O]^+^
							257.11658	C_16_H_17_O_3_	−2.493	[M + H − C_2_H_5_OH − H_2_O − C_4_H_8_]^+^
							253.15779	C_18_H_21_O	−3.562	[M + H − C_2_H_5_OH − CO − 2H_2_O − CH_2_]^+^
							251.17871	C_19_H_23_	−2.856	[M + H − C_2_H_5_OH − CO − H_2_O − O − H_2_O]^+^
							249.16325	C_19_H_21_	−2.116	[M + H − C_2_H_5_OH − 3H_2_O − CO]^+^
							239.17874	C_18_H_23_	−2.874	[M + H − C_2_H_5_OH − 2CO − 2H_2_O]^+^
							223.14754	C_17_H_19_	−2.631	[M + H − C_2_H_5_OH − 2CO − 2H_2_O − CH_4_]^+^
7,20-non-epoxy-kaurane diterpenoids	Henryin (**57**)	[M + H]^+^	393.22580	C_22_H_33_O_6_	−3.472	MS^2^[393.23]	375.21652	C_22_H_31_O_5_	−0.215	[M + H − H_2_O]^+^
							333.20602	C_20_H_29_O_4_	−0.048	[M + H − C_2_H_4_O_2_]^+^
						MS^3^[375.22]	357.20593	C_22_H_29_O_4_	−0.296	[M + H − 2H_2_O]^+^
							333.20541	C_20_H_29_O_4_	−1.878	[M + H − H_2_O − C_2_H_2_O]^+^
							315.19522	C_20_H_27_O_3_	−0.797	[M + H − H_2_O − C_2_H_4_O_2_]^+^
							297.18463	C_20_H_25_O_2_	−0.931	[M + H − H_2_O − C_2_H_4_O_2_ − H_2_O]^+^
							279.17404	C_20_H_23_O	−1.081	[M + H − H_2_O − C_2_H_4_O_2_ − 2H_2_O]^+^
							269.18973	C_19_H_25_O	−0.973	[M + H − H_2_O − C_2_H_4_O_2_ − H_2_O − CO]^+^
							267.17410	C_19_H_23_O	−0.905	[M + H − H_2_O − C_2_H_4_O_2_ − H_2_O − CH_2_O]^+^
							253.15826	C_18_H_21_O	−1.706	[M + H − H_2_O − C_2_H_4_O_2_ − H_2_O − CH_2_O − CH_2_]^+^
							251.17911	C_19_H_23_	−1.263	[M + H − H_2_O − C_2_H_4_O_2_ − 2H_2_O − CO]^+^
	Kamebakaurin (**44**)	[M + NH_4_]^+^	368.24252	C_20_H_34_O_5_N	−1.710	MS^2^[368.24]	351.21631	C_20_H_31_O_5_	−0.827	[M + NH_4_ − NH_3_]^+^
							333.20581	C_20_H_29_O_4_	−0.678	[M + NH_4_ − NH_3_ − H_2_O]^+^
							315.19519	C_20_H_27_O_3_	−0.892	[M + NH_4_ − NH_3_ − 2H_2_O]^+^
							297.18460	C_20_H_25_O_2_	−1.032	[M + NH_4_ − NH_3_ − 3H_2_O]^+^
							287.20023	C_19_H_27_O_2_	−1.137	[M + NH_4_ − NH_3_ − 2H_2_O − CO]^+^
							285.18460	C_19_H_25_O_2_	−1.075	[M + NH_4_ − NH_3_ − 2H_2_O − CH_2_O]^+^
							269.18939	C_19_H_25_O	−2.236	[M + NH_4_ − NH_3_ − 3H_2_O − CO]^+^
							267.17398	C_19_H_23_O	−1.355	[M + NH_4_ − NH_3_ − 3H_2_O − CH_2_O]^+^
						MS^3^[333.21]	315.19519	C_20_H_27_O_3_	−0.892	[M + NH_4_ − NH_3_ − 2H_2_O]^+^
							297.18454	C_20_H_25_O_2_	−1.233	[M + NH_4_ − NH_3_ − 3H_2_O]^+^
							279.17328	C_20_H_23_O	−3.804	[M + NH_4_ − NH_3_ − 4H_2_O]^+^
							251.17920	C_19_H_23_	−0.790	[M + NH_4_ − NH_3_ − 4H_2_O − CO]^+^
	Kamebanin (**52**)	[M + NH_4_]^+^	352.24734	C_20_H_34_O_4_N	−2.541	MS^2^[352.25]	335.22070	C_20_H_31_O_4_	−2.941	[M + NH_4_ − NH_3_]^+^
							317.21017	C_20_H_29_O_3_	−2.999	[M + NH_4_ − NH_3_ − H_2_O]^+^
						MS^3^[317.21]	299.19965	C_20_H_27_O_2_	−3.030	[M + NH_4_ − NH_3_ − 2H_2_O]^+^
							281.18921	C_20_H_25_O	−2.781	[M + NH_4_ − NH_3_ − 3H_2_O]^+^
							271.20486	C_19_H_27_O	−2.883	[M + NH_4_ − NH_3_ − 2H_2_O − CO]^+^
							253.19402	C_19_H_25_	−4.176	[M + NH_4_ − NH_3_ − 3H_2_O − CO]^+^
	1α,7α-dihydroxy-14β,20-diacetoxy-ent-kaur-16-en-15-one (**64**)	[M + NH_4_]^+^	452.26346	C_24_H_38_O_7_N	−1.811	MS^2^[452.26]	435.23743	C_24_H_35_O_7_	−0.689	[M + NH_4_ − NH_3_]^+^
							417.22687	C_24_H_33_O_6_	−0.708	[M + NH_4_ − NH_3_ − H_2_O]^+^
							375.21652	C_22_H_31_O_5_	−0.215	[M + NH_4_ − NH_3_ − H_2_O − C_2_H_2_O]^+^
						MS^3^[417.23]	375.21652	C_22_H_31_O_5_	−0.215	[M + NH_4_ − NH_3_ − H_2_O − C_2_H_2_O]^+^
							357.20592	C_22_H_29_O_4_	−0.324	[M + NH_4_ − NH_3_ − H_2_O − C_2_H_2_O − H_2_O]^+^
							339.19460	C_22_H_27_O_3_	−2.630	[M + NH_4_ − NH_3_ − H_2_O − C_2_H_2_O − 2H_2_O]^+^
							329.21146	C_21_H_29_O_3_	1.029	[M + NH_4_ − NH_3_ − H_2_O − C_2_H_2_O − H_2_O − CO]^+^
							315.19460	C_20_H_27_O_3_	−2.830	[M + NH_4_ − NH_3_ − H_2_O − C_2_H_2_O − H_2_O − C_2_H_2_O]^+^
							297.18420	C_20_H_25_O_2_	−2.280	[M + NH_4_ − NH_3_ − H_2_O − C_2_H_2_O − H_2_O − C_2_H_2_O − H_2_O]^+^
							279.17360	C_20_H_23_O	−2.730	[M + NH_4_ − NH_3_ − H_2_O − C_2_H_2_O − H_2_O − C_2_H_2_O − 2H_2_O]^+^
							269.19019	C_19_H_25_O	0.736	[M + NH_4_ − NH_3_ − H_2_O − C_2_H_2_O − H_2_O − C_2_H_2_O − H_2_O − CO]^+^
							267.17370	C_19_H_23_O	−2.590	[M + NH_4_ − NH_3_ − H_2_O − C_2_H_2_O − H_2_O − C_2_H_2_O − H_2_O − CH_2_O]^+^
							251.17940	C_19_H_23_	−0.230	[M + NH_4_ − NH_3_ − H_2_O − C_2_H_2_O − H_2_O − C_2_H_2_O − H_2_O − CO − H_2_O]^+^
	1α,14β-dihydroxy-7α,20-diacetoxy-ent-kaur-16-en-15-one (**74**)	[M + H]^+^	435.23727	C_24_H_35_O_7_	−1.057	MS^2^[435.24]	417.22702	C_24_H_33_O_6r_	−0.348	[M + H − H_2_O]^+^
						MS^3^[417.23]	375.21594	C_22_H_31_O_5_	−1.760	[M + H − H_2_O − C_2_H_2_O]^+^
							357.20505	C_22_H_29_O_4_	−2.760	[M + H − H_2_O − C_2_H_2_O − H_2_O]^+^
							339.19467	C_22_H_27_O_3_	−2.362	[M + H − H_2_O − C_2_H_2_O − 2H_2_O]^+^
							327.19464	C_21_H_27_O_3_	−2.540	[M + H − H_2_O − C_2_H_2_O − H_2_O − CH_2_O]^+^
							315.19461	C_20_H_27_O_3_	−2.732	[M + H − H_2_O − C_2_H_2_O − H_2_O − C_2_H_2_O]^+^
							297.18417	C_20_H_25_O_2_	−2.478	[M + H − H_2_O − C_2_H_2_O − H_2_O − C_2_H_2_O − H_2_O]^+^
							279.17363	C_20_H_23_O	−2.550	[M + H − H_2_O − C_2_H_2_O − H_2_O − C_2_H_2_O − 2H_2_O]^+^
							269.18918	C_19_H_25_O	−3.016	[M + H − H_2_O − C_2_H_2_O − H_2_O − C_2_H_2_O − H_2_O − CO]^+^
							267.17370	C_19_H_23_O	−2.590	[M + H − H_2_O − C_2_H_2_O − H_2_O − C_2_H_2_O − H_2_O − CH_2_O]^+^
							251.17890	C_19_H_23_	−2.099	[M + H − H_2_O − C_2_H_2_O − H_2_O − C_2_H_2_O − H_2_O − CO − H_2_O]^+^
	1α,7α,14β-trihydroxy-20-acetoxy-ent-kaur-15-one (**54**)	[M + NH_4_]^+^	412.26825	C_22_H_38_O_6_N	−2.703	MS^2^[412.27]	395.24252	C_22_H_35_O_6_	−0.747	[M + NH_4_ − NH_3_]^+^
							377.23126	C_22_H_33_O_5_	−2.626	[M + NH_4_ − NH_3_ − H_2_O]^+^
							359.22076	C_22_H_31_O_4_	−2.578	[M + NH_4_ − NH_3_ − 2H_2_O]^+^
							333.24158	C_21_H_33_O_3_	−2.525	[M + NH_4_ − NH_3_ − H_2_O − CO_2_]^+^
							315.23108	C_21_H_31_O_2_	−2.464	[M + NH_4_ − NH_3_ − H_2_O − CO_2_ − H_2_O]^+^
							273.22086	C_19_H_29_O	−1.581	[M + NH_4_ − NH_3_ − H_2_O − CO_2_ − H_2_O − C_2_H_2_O]^+^
							255.20996	C_19_H_27_	−3.007	[M + NH_4_ − NH_3_ − H_2_O − CO_2_ − H_2_O − C_2_H_2_O − H_2_O]^+^
						MS^3^[359.22]	341.21021	C_22_H_29_O_3_	−2.671	[M + NH_4_ − NH_3_ − 3H_2_O]^+^
							317.21024	C_20_H_29_O_3_	−2.778	[M + NH_4_ − NH_3_ − 2H_2_O − C_2_H_2_O]^+^
							299.19968	C_20_H_27_O_2_	−2.930	[M + NH_4_ − NH_3_ − 2H_2_O − C_2_H_2_O − H_2_O]^+^
							281.18918	C_20_H_25_O	−2.888	[M + NH_4_ − NH_3_ − 2H_2_O − C_2_H_2_O − 2H_2_O]^+^
							271.20483	C_19_H_27_O	−2.994	[M + NH_4_ − NH_3_ − 2H_2_O − C_2_H_2_O − H_2_O − CO]^+^
							253.19429	C_19_H_25_	−3.110	[M + NH_4_ − NH_3_ − 2H_2_O − C_2_H_2_O − H_2_O − CO − H_2_O]^+^
							225.16298	C_17_H_21_	−3.541	[M + NH_4_ − NH_3_ − 2H_2_O − C_2_H_2_O − H_2_O − CO − H_2_O − C_2_H_4_]^+^
							197.13176	C_15_H_17_	−3.638	[M + NH_4_ − NH_3_ − 2H_2_O − C_2_H_2_O − H_2_O − CO − H_2_O − 2C_2_H_4_]^+^
							169.10049	C_13_H_13_	−4.063	[M + NH_4_ − NH_3_ − 2H_2_O − C_2_H_2_O − H_2_O − CO − H_2_O − 3C_2_H_4_]^+^

Notes: “Elemental Comp.” denotes elemental composition.

**Table 2:** MS*^n^* key diagnostic ions and fragmentation pathways of the compounds identified from IEH.

Compound No.	t_R_/min	Selection	Measured Mass	Molecular Formula	Error (ppm)	MS*^n^* Key Diagnostic *m*/*z*	Type	Rel. Abund. (%)
**1** **	17.22	[M + NH_4_]^+^	382.22133	C_20_H_32_O_6_N	−2.837	MS^2^[382.22]: 365.19510; MS3[365.20]: 347.18460.	A	0.52
**2** *	20.84	[M + H]^+^	365.19547	C_20_H_29_O_6_	−1.082	MS^2^[365.20]: 347.18503; MS^3^[347.19]: 329.17453, 311.16391, 301.17957, 299.16394, 293.15350, 283.16901, 265.15848.	A	0.25
**3** **	27.05	[M + H]^+^	375.21602	C_22_H_31_O_5_	−1.547	MS^2^[375.22]: 357.20556, 315.19490, 297.18444, 269.18945, 251.17895.	A	1.73
**4** ***	27.66	[M + H]^+^	349.20021	C_20_H_29_O_5_	−2.121	MS^2^[349.20]: 331.18974, 249.16167; MS3[331.19]: 313.17917, 295.16859, 285.18444, 267.17357.	A	5.18
**5** *	39.30	[M + H]^+^	407.20526	C_22_H_31_O_7_	−2.873	MS^2^[407.20]: 389.19492; MS^3^[389.19]: 371.18428, 329.17390, 311.16335, 299.16343, 283.16840, 265.15780.	A	0.14
**6** ***	40.78	[M + NH_4_]^+^	364.21083	C_20_H_30_O_5_N	−2.799	MS^2^[364.21]: 347.18448, 329.17404, 311.16309; MS3[329.17]: 283.16878, 265.15810.	A	0.73
**7** **	43.97	[M + H]^+^	375.21566	C_22_H_31_O_5_	−2.507	MS^2^[375.22]: 357.20543, 315.19494; MS3[315.19]: 297.18438, 269.18949, 251.17889.	A	7.42
**8** ***	45.94	[M + H]^+^	315.19451	C_20_H_27_O_3_	−3.050	MS^2^[315.19]: 297.18423, 273.14837, 269.18931.	A	1.89
**9** ***	47.19	[M + H]^+^	331.18974	C_20_H_27_O_4_	−1.950	MS^2^[331.19]: 313.17943, 289.14290, 285.18441.	A	0.83
**10** ***	47.46	[M + H]^+^	317.21039	C_20_H_29_O_3_	−2.305	MS^2^[317.21]: 299.20008; MS3[299.20]: 281.18955, 271.20516, 253.19460.	A	6.67
**11** ***	48.74	[M + H]^+^	317.21009	C_20_H_29_O_3_	−3.251	MS^2^[317.21]: 299.20005, 281.18992, 275.16367.	A	2.71
**12** ***	49.27	[M + H]^+^	317.21021	C_20_H_29_O_3_	−2.873	MS^2^[317.21]: 299.20026, 281.18937, 275.16367.	A	3.62
**13** *	50.40	[M + H]^+^	375.2157	C_22_H_31_O_5_	−2.400	MS^2^[375.22]: 357.20512, 315.19457, 285.18404; MS^3^[315.19]: 297.18391, 287.19979, 269.18905, 251.17857.	A	0.04
**14** ***	52.83	[M + H]^+^	317.21036	C_20_H_29_O_3_	−2.400	MS^2^[317.21]: 299.19996; MS3[299.20]: 281.18937, 271.20510, 253.19460.	A	1.33
**15** ***	57.63	[M + H]^+^	357.20553	C_22_H_29_O_4_	−1.416	MS^2^[357.20]: 297.18432; MS3[297.18]: 255.13745, 241.12177.	A	0.36
**16** ***	61.95	[M + H]^+^	359.22089	C_22_H_31_O_4_	−2.216	MS^2^[359.22]: 299.19959, 281.18973.	A	6.67
**17** ***	64.90	[M + H]^+^	315.19463	C_20_H_27_O_3_	−2.669	MS^2^[315.19]: 297.18374, 273.14792, 269.18937.	A	4.48
**18** **	36.96	[M + NH_4_ − NH_3_ − H_2_O]^+^ [2M + NH_4_]^+^ [M + NH_4_]^+^	331.18956 714.41947 366.22681	C_20_H_27_O_4_ C_40_H_60_O_10_N C_20_H_32_O_5_N	−2.493 −2.384 −1.883	MS^2^[331.19]: 313.17921; MS3[313.18]: 295.16881, 285.18441, 277.15819, 267.17379, 249.16336.	B	2.78
**19** **	38.88	[M + NH_4_ − NH_3_ − H_2_O]^+^ [2M + NH_4_]^+^ [M + NH_4_]^+^	331.18992 714.41933 366.22655	C_20_H_27_O_4_ C_40_H_60_O_10_N C_20_H_32_O_5_N	−1.406 −2.580 −2.593	MS^2^[331.19]: 313.17952; MS3[313.18]: 295.16887, 285.18457, 277.15831, 267.17398, 249.16341.	B	3.09
**20** **	39.05	[M + NH_4_ − NH_3_ − H_2_O]^+^ [2M + NH_4_]^+^ [M + NH_4_]^+^	331.18983 714.41976 366.22674	C_20_H_27_O_4_ C_40_H_60_O_10_N C_20_H_32_O_5_N	−1.678 −1.978 −2.074	MS^2^[331.19]: 313.17964; MS3[313.18]: 295.16887, 285.18457, 277.15828, 267.17395, 249.16342.	B	3.55
**21** *	44.59	[M + NH_4_ − NH_3_ − CH_4_O]^+^ [2M + NH_4_]^+^ [M + NH_4_]^+^	331.18945 742.45083 380.24484	C_20_H_27_O_4_ C_42_H_64_O_10_N C_21_H_34_O_5_N	−2.826 −2.214 −2.603	MS^2^[331.19]: 313.17917; MS^3^[313.18]: 295.16855, 285.18420, 277.15793, 267.17355, 249.16306.	B	7.51
**22** *	45.36	[M + NH_4_ − NH_3_ − CH_4_O]^+^ [2M + NH_4_]^+^ [M + NH_4_]^+^	331.18976 742.45300 380.24214	C_20_H_27_O_4_ C_42_H_64_O_10_N C_21_H_34_O_5_N	−1.890 0.709 −2.655	MS^2^[331.19]: 313.17911; MS^3^[313.18]: 295.16855, 285.18414, 277.15796, 267.17365, 249.16313.	B	12.74
**23** *	52.03	[M + NH_4_ − NH_3_ − C_2_H_5_OH]^+^ [2M + NH_4_]^+^ [M + NH_4_]^+^	331.18979 770.48199 394.25772	C_20_H_27_O_4_ C_44_H_68_O_10_N C_22_H_36_O_5_N	−1.799 −2.315 −2.739	MS^2^[331.19]: 313.17914; MS^3^[313.18]: 295.16861, 285.18417, 277.15799, 267.17365, 249.16325.	B	0.23
**24** **	5.88	[M + NH_4_]^+^	368.24229	C_20_H_34_O_5_N	−2.334	MS^2^[368.24]: 351.21612, 333.20559, 315.19521, 297.18456.	C	0.63
**25** ***	8.69	[M + NH_4_]^+^	386.25285	C_20_H_36_O_6_N	−2.238	MS^2^[386.25]: 351.21587; MS3[351.22]: 333.20547, 315.19509, 297.18380.	C	1.74
**26** ***	14.88	[M + NH_4_]^+^	428.26331	C_22_H_38_O_7_N	−2.262	MS^2^[428.26]: 411.23732, 393.22664, 375.21614; MS3[375.22]: 333.20562, 315.19491, 297.18423.	C	8.31
**27** **	14.94	[M + NH_4_]^+^	410.25306	C_22_H_36_O_6_N	−1.595	MS^2^[410.25]: 357.20556, 315.19463, 297.18420; MS3[297.18]: 279.17416, 269.19025, 251.17904.	C	0.73
**28** ***	15.17	[M + NH_4_]^+^	532.31022	C_26_H_46_O_10_N	−2.636	MS^2^[532.31]: 515.28317, 335.22125, 317.20999, 299.19937.	C	0.34
**29** ***	15.46	[M + NH_4_]^+^	438.28408	C_24_H_40_O_6_N	−2.132	MS^2^[438.28]: 315.19450, 297.18444, 279.17431, 251.17872.	C	1.59
**30** **	15.49	[M + NH_4_]^+^	410.25254	C_22_H_36_O_6_N	−2.862	MS^2^[410.25]: 357.20534, 315.19443, 297.18438.	C	0.60
**31** ***	16.61	[M + H]^+^	333.20352	C_20_H_29_O_4_	−2.516	MS^2^[333.20]: 315.19342, 297.18295.	C	8.58
**32** **	16.87	[M + NH_4_]^+^	368.24229	C_20_H_34_O_5_N	−2.334	MS^2^[368.24]: 351.21615, 333.20562, 279.17425; MS3[333.20]: 315.19497, 297.18441.	C	3.53
**33** ***	17.00	[M + NH_4_]^+^	426.24749	C_22_H_36_O_7_N	−2.672	MS^2^[426.25]: 391.21082, 373.20026, 331.18989, 285.18393, 267.17425; MS3[373.20]: 313.18025, 295.16862.	C	0.49
**34** ***	17.42	[M + NH_4_]^+^	428.2631	C_22_H_38_O_7_N	−2.753	MS^2^[428.26]: 411.23711, 393.22609, 375.21553, 333.20522, 297.18420; MS3[333.20]: 315.19473.	C	1.17
**35** **	18.01	[M + H]^+^	351.21587	C_20_H_31_O_5_	−2.080	MS^2^[351.22]: 333.20374, 315.19339; MS3[315.19]: 297.18280, 279.17239.	C	1.45
**36** **	18.53	[M + NH_4_]^+^	368.24247	C_20_H_34_O_5_N	−1.846	MS^2^[368.24]: 351.21560, 333.20547; MS3[333.20]: 315.19485.	C	0.52
**37** **	18.75	[M + NH_4_]^+^	368.24235	C_20_H_34_O_5_N	−2.171	MS^2^[368.24]: 351.21602, 333.20550, 315.19500; MS3[315.19]: 297.18441.	C	1.06
**38** ***	18.75	[M + NH_4_]^+^	386.25288	C_20_H_36_O_6_N	−2.160	MS^2^[386.25]: 351.21587, 333.20510, 315.19442.	C	0.13
**39** **	19.49	[M + H]^+^	335.21927	C_20_H_31_O_4_	−2.416	MS^2^[335.22]: 317.20923, 299.19867; MS3[299.20]: 281.18765, 271.20383, 253.19353.	C	0.93
**40** **	19.88	[M + NH_4_]^+^	368.24244	C_20_H_34_O_5_N	−1.927	MS^2^[368.24]: 333.20553; MS3[333.21]: 315.19494, 297.18429, 279.17358.	C	3.25
**41** ***	19.98	[M + NH_4_]^+^	366.22659	C_20_H_32_O_5_N	−2.484	MS^2^[366.22]: 331.18974; MS3[331.19]: 313.17918, 295.16875.	C	2.28
**42** ***	20.17	[M + NH_4_]^+^	428.26328	C_22_H_38_O_7_N	−2.332	MS^2^[428.26]: 411.23744, 393.22692, 375.21614, 333.20550, 315.19485; MS3[315.19]: 297.18405.	C	1.05
**43** **	20.2	[M + NH_4_]^+^	368.24238	C_20_H_34_O_5_N	−2.090	MS^2^[368.24]: 351.21599, 333.20534; MS3[333.21]: 315.19519, 297.18405.	C	0.59
**44** *	21.36	[M + NH_4_]^+^	368.24252	C_20_H_34_O_5_N	−1.710	MS^2^[368.24]: 351.21631, 333.20581, 315.19519, 269.18939, 267.17398; MS^3^[333.21]: 315.19519, 297.18454, 279.17328, 251.17920.	C	29.15
**45** ***	21.58	[M + NH_4_]^+^	470.27329	C_24_H_40_O_8_N	−3.304	MS^2^[470.27]: 453.24679, 435.23659; MS3[435.23]: 393.22571.	C	1.06
**46** **	21.71	[M + NH_4_]^+^	352.24548	C_20_H_34_O_4_N	−2.755	MS^2^[352.25]: 335.21936, 317.20898; MS3[317.21]: 299.19855, 281.18893.	C	1.08
**47** ***	21.84	[M + H]^+^	333.20364	C_20_H_29_O_4_	−2.396	MS^2^[333.20]: 315.19330, 297.18286.	C	4.81
**48** ***	22.13	[M + NH_4_]^+^	370.25798	C_20_H_36_O_5_N	−2.214	MS^2^[370.26]: 335.22113; MS3[335.22]: 317.21039, 299.19996.	C	10.83
**49** ***	22.55	[M + NH_4_]^+^	426.24749	C_22_H_36_O_7_N	−2.672	MS^2^[426.25]: 391.21046, 373.19947, 285.18457; MS3[391.21]: 331.18959, 313.17888, 295.16872.	C	0.76
**50** **	22.94	[M + NH_4_]^+^	368.24223	C_20_H_34_O_5_N	−2.497	MS^2^[368.24]: 351.21578, 297.18388; MS3[351.21]: 333.20457, 315.19500.	C	0.69
**51** **	24.98	[M + NH_4_]^+^	410.25300	C_22_H_36_O_6_N	−1.741	MS^2^[410.25]: 375.21587, 357.20543 269.18925; MS3[375.22]: 357.20540, 315.19482, 297.18432, 279.17401, 251.17931.	C	100.00
**52** *	25.94	[M + NH_4_]^+^	352.24734	C_20_H_34_O_4_N	−2.541	MS^2^[352.25]: 335.22070, 317.21017; MS^3^[317.21]: 299.19965, 281.18921, 271.20486, 253.19402.	C	30.28
**53** **	26.38	[M + NH_4_]^+^	368.24235	C_20_H_34_O_5_N	−2.171	MS^2^[368.24]: 351.21581, 333.20504; MS3[333.21]: 315.19534, 297.18381.	C	4.58
**54** *	26.54	[M + NH_4_]^+^	412.26825	C_22_H_38_O_6_N	−2.703	MS^2^[412.27]: 395.24252, 377.23126, 359.22076; MS^3^[359.22]: 341.21021, 317.21024, 299.19968, 281.18918, 271.20483, 253.19429.	C	27.33
**55** ***	26.82	[M + NH_4_]^+^	354.26331	C_20_H_36_O_4_N	−1.623	MS^2^[354.26]: 337.23686, 319.22630; MS3[319.23]: 301.21562, 283.20509.	C	4.05
**56** ***	27.17	[M + NH_4_]^+^	470.27329	C_24_H_40_O_8_N	−3.304	MS^2^[470.27]: 453.24699, 393.22615, 333.20528, 315.19485.	C	1.17
**57** *	27.27	[M + H]^+^	393.22580	C_22_H_33_O_6_	−3.472	MS^2^[393.23]: 375.21652, 333.20602; MS^3^[375.22]: 357.20593, 333.20541, 315.19522, 297.18463, 279.17404, 269.18973, 267.17410, 251.17911.	C	0.82
**58** ***	27.37	[M + NH_4_]^+^	454.27852	C_24_H_40_O_7_N	−3.102	MS^2^[454.28]: 437.25237, 419.24142, 377.23184, 359.22109, 317.21100, 299.20005, 281.18955.	C	4.34
**59** **	27.88	[M + NH_4_]^+^	410.25263	C_22_H_36_O_6_N	−2.643	MS^2^[410.25]: 375.21556, 315.19500; MS3[375.22]: 357.20546, 297.18429, 279.17389.	C	0.85
**60** **	29.06	[M + NH_4_]^+^	410.25248	C_22_H_36_O_6_N	−3.009	MS^2^[410.25]: 375.21553; MS3[375.22]: 357.20534, 315.19494, 297.18435, 279.17379, 269.18943, 251.17881.	C	9.96
**61** ***	29.44	[M + NH_4_]^+^	398.25248	C_21_H_36_O_6_N	−3.099	MS^2^[393.23]: 381.22606, 363.21569, 345.20565; MS3[363.22]: 331.18940.	C	0.79
**62** **	29.98	[M + H]^+^	351.21381	C_20_H_31_O_5_	−2.791	MS^2^[351.21]: 333.20367, 315.19324; MS3[333.20]: 297.18295, 279.17239.	C	0.79
**63** ***	32.20	[M + NH_4_]^+^	484.28895	C_25_H_42_O_8_N	−3.188	MS^2^[484.29]: 467.26253, 449.25191.	C	1.87
**64** *	33.20	[M + NH_4_]^+^	452.26346	C_24_H_38_O_7_N	−1.811	MS^2^[452.26]: 435.23743, 417.22687; MS^3^[417.23]: 375.21652, 357.20592, 339.19460, 329.21146, 315.19460, 297.18420, 279.17360, 269.19019, 267.17370, 251.17940.	C	112.95
**65** **	34.44	[M + NH_4_]^+^	394.25592	C_22_H_36_O_5_N	−2.880	MS^2^[394.26]: 377.23016, 359.21915; MS3[359.22]: 317.20892, 299.19861.	C	1.13
**66** **	34.91	[M + NH_4_]^+^	410.2526	C_22_H_36_O_6_N	−2.716	MS^2^[410.25]: 375.21566, 251.17864; MS3[375.22]: 357.20534, 315.19497, 297.18441, 279.17376, 269.18974.	C	43.67
**67** **	35.51	[M + NH_4_]^+^	412.26826	C_22_H_38_O_6_N	−2.675	MS^2^[412.27]: 395.24182, 377.23157, 317.21039, 281.18854; MS3[317.21]: 299.19983.	C	5.31
**68** **	36.50	[M + NH_4_]^+^	452.26065	C_24_H_38_O_7_N	−3.629	MS^2^[452.26]: 435.23636, 375.21371; MS3[375.22]: 357.20522, 315.19531, 297.18426.	C	61.69
**69** **	39.50	[M + NH_4_]^+^	452.26309	C_24_H_38_O_7_N	−2.629	MS^2^[452.26]: 435.23674; MS3[435.23]: 417.22682, 375.21566, 357.20528, 315.19466, 297.18423, 279.17355.	C	17.04
**70** ***	39.66	[M + H]^+^	333.20364	C_20_H_29_O_4_	−2.396	MS^2^[333.20]: 315.19330; MS3[315.19]: 297.18304.	C	3.83
**71** ***	40.42	[M + H]^+^	303.23123	C_20_H_31_O_2_	−2.067	MS^2^[303.23]: 285.22079, 267.21014; MS3[267.21]: 239.17843, 225.16322, 211.14779, 197.13196.	C	0.89
**72** **	41.55	[M + H]^+^	435.23649	C_24_H_35_O_7_	−2.849	MS^2^[435.24]: 417.22607; MS^3^[417.23]: 357.20556, 297.18494, 279.17379.	C	1.02
**73** ***	40.48	[M + NH_4_]^+^	454.27882	C_24_H_40_O_7_N	−2.441	MS^2^[454.28]: 437.25179, 419.24145, 359.22086.	C	0.58
**74** *	41.00	[M + H]^+^	435.23727	C_24_H_35_O_7_	−1.057	MS^2^[435.24]: 417.22702; MS^3^[417.23]: 375.21594, 357.20505, 339.19467, 327.19464, 315.19461, 297.18417, 279.17363, 269.18918, 267.17370, 251.17890.	C	17.27
**75** **	41.36	[M + NH_4_]^+^	352.24737	C_20_H_34_O_4_N	−2.456	MS^2^[352.25]: 335.22095, 317.21042, 271.20461; MS3[317.21]: 299.19977, 281.18995, 253.19451.	C	6.62
**76** ***	41.64	[M + NH_4_]^+^	366.22662	C_20_H_32_O_5_N	−2.402	MS^2^[366.22]: 331.18977; MS3[331.19]: 313.17903, 295.16782.	C	1.62
**77** **	42.23	[M + NH_4_]^+^	452.26306	C_24_H_38_O_7_N	−2.695	MS^2^[452.16]: 435.23665, 375.21587, 357.20531, 279.17389; MS3[375.23]: 315.19473, 297.18417.	C	13.72
**78** ***	42.77	[M + NH_4_]^+^	354.26318	C_20_H_36_O_4_N	−1.990	MS^2^[354.26]: 337.23634, 319.22593; MS3[319.22]: 301.21605, 283.20509.	C	0.41
**79** ***	44.14	[M + NH_4_]^+^	496.28898	C_26_H_42_O_8_N	−3.050	MS^2^[496.29]: 479.26233, 461.25216, 419.24148, 401.23123, 359.22055.	C	9.41
**80** ***	44.66	[M + NH_4_]^+^	350.23182	C_20_H_32_O_4_N	−2.184	MS^2^[350.23]: 333.20544; MS3[333.21]: 315.19488, 297.18478.	C	2.76
**81** ***	44.87	[M + NH_4_]^+^	366.22671	C_20_H_32_O_5_N	−2.156	MS^2^[366.22]: 331.18974; MS3[331.19]: 313.17924, 295.16798.	C	20.89
**82** ***	45.25	[M + NH_4_]^+^	392.24216	C_22_H_34_O_5_N	−2.523	MS^2^[392.24]: 375.21563; MS3[375.22]: 357.20531, 333.20544, 315.19488, 297.18429, 279.17373, 269.18937, 251.17884.	C	7.77
**83** **	46.26	[M + NH_4_]^+^	368.24235	C_20_H_34_O_5_N	−2.171	MS^2^[368.24]: 351.21593, 333.20544; MS3[333.21]: 315.19482.	C	0.77
**84** **	46.72	[M + NH_4_]^+^	352.24746	C_20_H_34_O_4_N	−2.200	MS^2^[352.25]: 335.22110, 317.21064; MS3[317.21]: 299.20005, 281.18915, 271.20583.	C	0.72
**85** ***	47.00	[M + NH_4_]^+^	450.24743	C_24_H_36_O_7_N	−2.663	MS^2^[450.25]: 433.22113; MS3[433.22]: 391.21061, 373.20002, 355.18939, 331.18965, 313.17943, 295.16887, 277.15749.	C	7.23
**86** ***	48.31	[M + NH_4_]^+^	496.28901	C_26_H_42_O_8_N	−2.990	MS^2^[496.29]: 479.26265, 461.25216, 401.23098, 377.23126, 317.21039; MS3[461.25]: 419.24182, 359.22113, 341.21027, 299.19983, 281.18928.	C	36.42
**87** ***	48.64	[M + H]^+^	421.25696	C_24_H_37_O_6_	−3.573	MS^2^[421.25]: 403.24644, 361.23667, 343.22547, 283.20461; MS3[403.24]: 265.19429.	C	1.72
**88** ***	50.46	[M + NH_4_]^+^	496.28953	C_26_H_42_O_8_N	−1.942	MS^2^[496.29]: 479.26210, 461.25216, 419.24179, 401.23119, 359.22073.	C	1.01
**89** ***	52.08	[M + NH_4_]^+^	408.23703	C_22_H_34_O_6_N	−2.533	MS^2^[408.24]: 373.20011; MS3[373.20]: 355.18977, 331.18980, 313.17930, 295.16878, 277.15831, 267.17386, 249.16344.	C	4.93
**90** ***	52.86	[M + H]^+^	479.26214	C_26_H_39_O_8_	−3.765	MS^2^[479.26]: 461.25185; MS3[461.25]: 401.23077, 341.21002, 323.20026, 281.18915, 263.17945.	C	1.64
**91** ***	52.93	[M + H]^+^	771.46547	C_44_H_67_O_11_	−3.006	MS^2^[771.46]: 753.45439, 735.44527, 717.43462, 675.42416, 657.41339, 597.39227, 579.38236, 561.37219; MS3[657.41]: 639.40251, 393.22617.	C	1.49
**92** ***	54.29	[M + H]^+^	785.44552	C_44_H_65_O_12_	−1.953	MS^2^[785.44]: 767.43420, 707.41333, 671.39234, 661.40834, 643.39745, 375.21602, 315.19476, 297.18395; MS3[767.43]: 749.42453, 689.40134, 393.22684, 279.17331.	C	4.15
**93** **	55.09	[M + NH_4_]^+^	394.25623	C_22_H_36_O_5_N	−2.570	MS^2^[394.26]: 359.21933, 299.19839.	C	0.31
**94** ***	56.96	[M + NH_4_]^+^	538.2992	C_28_H_44_O_9_N	−3.452	MS2[538.30]: 461.25213; MS3[461.25]: 419.24093, 401.23022, 359.22049, 341.21008, 299.19983, 281.18912.	C	10.96

Notes: * Confirmed by comparing with reference compounds; ** compounds have been reported in the literature; *** potential new ent−kaurane diterpenoids. In the “type” column, A denotes bridgehead-unsubstituted 7,20-epoxy-ent-kaurane diterpenoids; B denotes bridgehead-substituted 7,20-epoxy-ent-kaurane diterpenoids; C denotes 7,20-non-epoxy-kaurane diterpenoids. “Rel. Abund.” denotes relative abundance, calculated as the percentage of a compound’s peak area relative to the maximum peak area in the TIC.

## Data Availability

Data are contained within the article and Supplementary Materials.

## Supplementary Materials

The following supporting information can be downloaded from https://www.mdpi.com/article/10.3390/molecules31020317/s1: Table S1: MS*^n^* data and fragmentation pathways of the compounds identified from IEH.
