# Supplementary material for: Characterization and Identification of the Ent-Kaurane Diterpenoids in Isodonis Excisoidis Herba Using UHPLC-LTQ-Orbitrap-MS
Source: Molecules. 2026 Jan 16;31(2):317. doi: 10.3390/molecules31020317 (PMC12844365; doi:10.3390/molecules31020317)
Supplement: Supplementary file 1 [file molecules-31-00317-s001.zip › molecules-4042253-supplementary.pdf]

Table S1. MS^n data and fragmentation pathways of the compounds identified from IEH.

Compound No.	t_R/min	Selection	Measured Mass	Molecular Formula	Error (ppm)	MS^n *m/z*	Type	Rel. Abund. (%)
**1** **	17.22	$[M + NH_4]^+$	382.22133	$C_{20}H_{32}O_6N$	−2.837	MS^2[382.22]: **365.19510**; MS^3[365.20]: **347.18460**.	A	0.52
**2** *	20.84	$[M + H]^+$	365.19547	$C_{20}H_{29}O_6$	−1.082	MS^2[365.20]: **347.18503**; MS^3[347.19]: **329.17453**, **311.16391**, **301.17957**, **299.16394**, **293.15350**, **283.16901**, 281.15338, 273.18451, **265.15848**, 255.17409, 247.14769, 237.16335.	A	0.25
**3** **	27.05	$[M + H]^+$	375.21602	$C_{22}H_{31}O_5$	−1.547	MS^2[375.22]: **357.20556**, 339.19511, **315.19490**, **297.18444**, **269.18945**, **251.17895**; MS^3[339.19]: 279.17384	A	1.73
**4** ***	27.66	$[M + H]^+$	349.20021	$C_{20}H_{29}O_5$	−2.121	MS^2[349.20]: **331.18974**, 301.17939, 271.16872, **249.16167**, 239.17890; MS^3[331.19]: **313.17917**, 301.17939, **295.16859**, **285.18444**, 283.16881, **267.17357**.	A	5.18
**5** *	39.30	$[M + H]^+$	407.20526	$C_{22}H_{31}O_7$	−2.873	MS^2[407.20]: **389.19492**, 347.18422; MS^3[389.19]: **371.18428**, 361.19980, 343.18951, **329.17390**, **311.16335**, 301.17896, **299.16343**, 293.15269, **283.16840**, 281.15285, **265.15780**, 247.14750, 237.16311, 223.14750.	A	0.14
**6** ***	40.78	$[M + NH_4]^+$	364.21083	$C_{20}H_{30}O_5N$	−2.799	MS^2[364.21]: **347.18448**, **329.17404**, **311.16309**; MS^3[329.17]: **283.16878**, **265.15810**, 237.16313.	A	0.73
**7** **	43.97	$[M + H]^+$	375.21566	$C_{22}H_{31}O_5$	−2.507	MS^2[375.22]: **357.20543**, 339.19466, **315.19494**, 267.17401, 241.19451; MS^3[315.19]: **297.18438**, 287.20026, 279.17382, **269.18949**, 255.13783, **251.17889**.	A	7.42
**8** ***	45.94	$[M + H]^+$	315.19451	$C_{20}H_{27}O_3$	−3.050	MS^2[315.19]: **297.18423**, **273.14837**, **269.18931**; MS^3[297.18]: 279.17376, 255.13733, 237.12663, 229.12159.	A	1.89
**9** ***	47.19	$[M + H]^+$	331.18974	$C_{20}H_{27}O_4$	−1.950	MS^2[331.19]: **313.17943**, 295.16887, **289.14290**, **285.18441**; MS^3[289.14]: 243.13745.	A	0.83
**10** ***	47.46	$[M + H]^+$	317.21039	$C_{20}H_{29}O_3$	−2.305	MS^2[317.21]: **299.20008**, 275.16364, 263.17893, 213.12692; MS^3[299.20]: **281.18955**, **271.20516**, 263.17878, **253.19460**.	A	6.67
**11** ***	48.74	$[M + H]^+$	317.21009	$C_{20}H_{29}O_3$	−3.251	MS^2[317.21]: **299.20005**, **281.18992**, **275.16367**, 257.18940, 239.14250, 229.12171, 217.12173, 203.10606; MS^3[203.10]: 175.07479, 161.05914, 133.06433.	A	2.71
**12** ***	49.27	$[M + H]^+$	317.21021	$C_{20}H_{29}O_3$	−2.873	MS^2[317.21]: **299.20026**, **281.18937**, **275.16367**, 257.15305, 243.13751, 225.12680, 203.10618, 187.11123; MS^3[299.20]: 239.14262, 183.07987, 163.07486, 145.06436.	A	3.62
**13** *	50.40	$[M + H]^+$	375.2157	$C_{22}H_{31}O_5$	−2.400	MS^2[375.22]: **357.20512**, 329.17385, **315.19457**, **285.18404**, 267.17347; MS^3[315.19]: **297.18391**, **287.19979**, 279.17335, **269.18905**, 255.13706, **251.17857**, 241.12133, 223.14756, 195.11619, 149.13191, 147.11626, 121.10061.	A	0.04

**14** ***	52.83	$[M + H]^+$	317.21036	$C_{20}H_{29}O_3$	−2.400	MS²[317.21]: **299.19996**; MS³[299.20]: **281.18937**, **271.20510**, 263.17933, **253.19460**.	A	1.33
**15** ***	57.63	$[M + H]^+$	357.20553	$C_{22}H_{29}O_4$	−1.416	MS²[357.20]: **297.18432**; MS³[297.18]: **255.13745**, **241.12177**, 229.12180.	A	0.36
**16** ***	61.95	$[M + H]^+$	359.22089	$C_{22}H_{31}O_4$	−2.216	MS²[359.22]: 317.17420, **299.19959**, **281.18973**, 229.12177, 213.12683; MS³[317.17]: 257.15324, 239.14251, 221.13174, 187.07478, 171.07989.	A	6.67
**17** ***	64.90	$[M + H]^+$	315.19463	$C_{20}H_{27}O_3$	−2.669	MS²[315.19]: **297.18374**, 287.19993, **273.14792**, **269.18937**, 245.15289; MS³[269.19]: 227.14251, 199.11114, 185.09555, 175.11126, 157.06419.	A	4.48
**18** **	36.96	$[M + NH_4 - NH_3 - H_2O]^+$<br>$[2M + NH_4]^+$<br>$[M + NH_4]^+$	331.18956<br>714.41947<br>366.22681	$C_{20}H_{27}O_4$<br>$C_{40}H_{60}O_{10}N$<br>$C_{20}H_{32}O_5N$	−2.493<br>−2.384<br>−1.883	MS²[331.19]: **313.17921**, 287.16416, 211.07468; MS³[313.18]: **295.16881**, **285.18441**, **277.15819**, 269.18940, **267.17379**, **249.16336**, 239.17906, 223.14834.	B	2.78
**19** **	38.88	$[M + NH_4 - NH_3 - H_2O]^+$<br>$[2M + NH_4]^+$<br>$[M + NH_4]^+$	331.18992<br>714.41933<br>366.22655	$C_{20}H_{27}O_4$<br>$C_{40}H_{60}O_{10}N$<br>$C_{20}H_{32}O_5N$	−1.406<br>−2.580<br>−2.593	MS²[331.19]: **313.17952**, 303.19528, 287.16389, 283.16918, 211.07499; MS³[313.18]: **295.16887**, **285.18457**, **277.15831**, 269.18952, **267.17398**, 259.14769, 257.11668, 253.15830, 251.17912, **249.16341**, 239.17904, 223.14762.	B	3.09
**20** **	39.05	$[M + NH_4 - NH_3 - H_2O]^+$<br>$[2M + NH_4]^+$<br>$[M + NH_4]^+$	331.18983<br>714.41976<br>366.22674	$C_{20}H_{27}O_4$<br>$C_{40}H_{60}O_{10}N$<br>$C_{20}H_{32}O_5N$	−1.678<br>−1.978<br>−2.074	MS²[331.19]: **313.17964**, 303.19534, 287.16407, 283.16893, 211.07508; MS³[313.18]: **295.16887**, **285.18457**, **277.15828**, 269.18952, **267.17395**, 259.14754, 257.11674, 253.15822, 251.17901, **249.16342**, 239.17900, 223.14770, 209.13203.	B	3.55
**21** *	44.59	$[M + NH_4 - NH_3 - CH_4O]^+$<br>$[2M + NH_4]^+$<br>$[M + NH_4]^+$	331.18945<br>742.45083<br>380.24484	$C_{20}H_{27}O_4$<br>$C_{42}H_{64}O_{10}N$<br>$C_{21}H_{34}O_5N$	−2.826<br>−2.214<br>−2.603	MS²[331.19]: **313.17917**, 303.19489, 287.16333, 283.16837, 211.07478; MS³[313.18]: **295.16855**, **285.18420**, **277.15793**, 269.18909, **267.17355**, 259.14761, 257.11627, 253.15836, 251.17877, **249.16306**, 239.17873, 223.14755.	B	7.51
**22** *	45.36	$[M + NH_4 - NH_3 - CH_4O]^+$<br>$[2M + NH_4]^+$<br>$[M + NH_4]^+$	331.18976<br>742.45300<br>380.24214	$C_{20}H_{27}O_4$<br>$C_{42}H_{64}O_{10}N$<br>$C_{21}H_{34}O_5N$	1.890<br>0.709<br>−2.655	MS²[331.19]: **313.17911**, 303.19470, 287.16345, 283.16861, 211.07480; MS³[313.18]: **295.16855**, **285.18414**, **277.15796**, 269.18924, **267.17365**, 259.14758, 257.11649, 253.15797, 251.17863, **249.16313**, 239.17865, 223.14743.	B	12.74
**23** *	52.03	$[M + NH_4 - NH_3 - C_2H_5OH]^+$<br>$[2M + NH_4]^+$<br>$[M + NH_4]^+$	331.18979<br>770.48199<br>394.25772	$C_{20}H_{27}O_4$<br>$C_{44}H_{68}O_{10}N$<br>$C_{22}H_{36}O_5N$	−1.799<br>−2.315<br>−2.739	MS²[331.19]: **313.17914**, 303.19507, 287.16364, 283.16855, 211.07483; MS³[313.18]: **295.16861**, **285.18417**, **277.15799**, 269.18939, **267.17365**, 259.14746, 257.11658, 253.15779, 251.17871, **249.16325**, 239.17874, 223.14754.	B	0.23

**24** **	5.88	$[M + NH_4]^+$	368.24229	$C_{20}H_{34}O_5N$	−2.334	MS²[368.24]: **351.21612**, **333.20559**, **315.19521**, **297.18456**.	C	0.63
**25** ***	8.69	$[M + NH_4]^+$	386.25285	$C_{20}H_{36}O_6N$	−2.238	MS²[386.25]: 369.22631, **351.21587**, 321.20565; MS³[351.22]: **333.20547**, **315.19509**, 303.19583, **297.18380**, 285.18466.	C	1.74
**26** ***	14.88	$[M + NH_4]^+$	428.26331	$C_{22}H_{38}O_7N$	−2.262	MS²[428.26]: **411.23732**, **393.22664**, **375.21614**, 363.21611, 349.23680; MS³[375.22]: 357.20540, **333.20562**, **315.19491**, **297.18423**, 287.20017, 285.18426, 279.17367, 269.18928, 267.17389, 257.18943, 251.17895, 239.17922.	C	8.31
**27** **	14.94	$[M + NH_4]^+$	410.25306	$C_{22}H_{36}O_6N$	−1.595	MS²[410.25]: **357.20556**, **315.19463**, **297.18420**; MS³[297.18]: **279.17416**, **269.19025**, **251.17904**.	C	0.73
**28** ***	15.17	$[M + NH_4]^+$	532.31022	$C_{26}H_{46}O_{10}N$	−2.636	MS²[532.31]: **515.28317**, **335.22125**, **317.20999**, **299.19937**.	C	0.34
**29** ***	15.46	$[M + NH_4]^+$	438.28408	$C_{24}H_{40}O_6N$	−2.132	MS²[438.28]: **315.19450**, **297.18444**, **279.17431**, **251.17872**.	C	1.59
**30** **	15.49	$[M + NH_4]^+$	410.25254	$C_{22}H_{36}O_6N$	−2.862	MS²[410.25]: **357.20534**, **315.19443**, **297.18438**.	C	0.60
**31** ***	16.61	$[M + H]^+$	333.20352	$C_{20}H_{29}O_4$	−2.516	MS²[333.20]: **315.19342**, **297.18295**, 287.19846; MS³[297.18]: 279.17230, 269.18820, 261.16199, 251.17752.	C	8.58
**32** **	16.87	$[M + NH_4]^+$	368.24229	$C_{20}H_{34}O_5N$	−2.334	MS²[368.24]: **351.21615**, **333.20562**, 303.19531, 287.19990, 285.18399, **279.17425**, 267.17389; MS³[333.20]: **315.19497**, **297.18441**.	C	3.53
**33** ***	17.00	$[M + NH_4]^+$	426.24749	$C_{22}H_{36}O_7N$	−2.672	MS²[426.25]: **391.21082**, **373.20026**, 347.22123, **331.18989**, **285.18393**, 269.18998, **267.17425**; MS³[373.20]: 355.18977, **313.18025**, **295.16862**.	C	0.49
**34** ***	17.42	$[M + NH_4]^+$	428.2631	$C_{22}H_{38}O_7N$	−2.753	MS²[428.26]: **411.23711**, **393.22609**, **375.21553**, 363.21502, **333.20522**, 303.19470, **297.18420**, 267.17340; MS³[333.20]: **315.19473**, 285.18417.	C	1.17
**35** **	18.01	$[M + H]^+$	351.21587	$C_{20}H_{31}O_5$	−2.080	MS²[351.22]: **333.20374**, **315.19339**; MS³[315.19]: **297.18280**, **279.17239**, 269.18765, 251.17738.	C	1.45
**36** **	18.53	$[M + NH_4]^+$	368.24247	$C_{20}H_{34}O_5N$	−1.846	MS²[368.24]: **351.21560**, **333.20547**, 269.18964; MS³[333.20]: **315.19485**.	C	0.52
**37** **	18.75	$[M + NH_4]^+$	368.24235	$C_{20}H_{34}O_5N$	−2.171	MS²[368.24]: **351.21602**, **333.20550**, **315.19500**, 269.18874; MS³[315.19]: **297.18441**.	C	1.06
**38** ***	18.75	$[M + NH_4]^+$	386.25288	$C_{20}H_{36}O_6N$	−2.160	MS²[386.25]: 369.22631, **351.21587**, **333.20510**, **315.19442**, 303.19492.	C	0.13
**39** **	19.49	$[M + H]^+$	335.21927	$C_{20}H_{31}O_4$	−2.416	MS²[335.22]: **317.20923**, **299.19867**, 289.21429; MS³[299.20]: **281.18765**, **271.20383**, **253.19353**.	C	0.93
**40** **	19.88	$[M + NH_4]^+$	368.24244	$C_{20}H_{34}O_5N$	−1.927	MS²[368.24]: 350.23204, **333.20553**; MS³[333.21]: **315.19494**, **297.18429**, 287.20026, 285.18426, **279.17358**, 269.18952, 251.17901.	C	3.25
**41** ***	19.98	$[M + NH_4]^+$	366.22659	$C_{20}H_{32}O_5N$	−2.484	MS²[366.22]: 348.21616, **331.18974**; MS³[331.19]: **313.17918**, 301.17943, **295.16875**, 285.18396, 267.17425.	C	2.28

**42** ***	20.17	$[M + NH_4]^+$	428.26328	$C_{22}H_{38}O_7N$	−2.332	MS^2[428.26]: **411.23744**, **393.22692**, **375.21614**, 363.21601, 357.20476, **333.20550**, **315.19485**; MS^3[315.19]: **297.18405**.	C	1.05
**43** **	20.2	$[M + NH_4]^+$	368.24238	$C_{20}H_{34}O_5N$	−2.090	MS^2[368.24]: **351.21599**, **333.20534**; MS^3[333.21]: **315.19519**, **297.18405**.	C	0.59
**44** *	21.36	$[M + NH_4]^+$	368.24252	$C_{20}H_{34}O_5N$	−1.710	MS^2[368.24]: **351.21631**, **333.20581**, **315.19519**, 297.18460, 287.20023, 285.18460, **269.18939**, **267.17398**; MS^3[333.21]: **315.19519, 297.18454**, **279.17328**, **251.17920**.	C	29.15
**45** ***	21.58	$[M + NH_4]^+$	470.27329	$C_{24}H_{40}O_8N$	−3.304	MS^2[470.27]: **453.24679**, **435.23659**; MS^3[435.23]: **393.22571**.	C	1.06
**46** **	21.71	$[M + NH_4]^+$	352.24548	$C_{20}H_{34}O_4N$	−2.755	MS^2[352.25]: **335.21936**, **317.20898**; MS^3[317.21]: **299.19855**, **281.18893**.	C	1.08
**47** ***	21.84	$[M + H]^+$	333.20364	$C_{20}H_{29}O_4$	−2.396	MS^2[333.20]: **315.19330**, **297.18286**, 287.19846; MS^3[297.18]: 279.17233, 269.18799, 261.16205, 251.17764.	C	4.81
**48** ***	22.13	$[M + NH_4]^+$	370.25798	$C_{20}H_{36}O_5N$	−2.214	MS^2[370.26]: 353.23166, **335.22113**, 287.20005, 269.18937; MS^3[335.22]: **317.21039**, **299.19996**, 289.21532, 281.18940, 271.20491.	C	10.83
**49** ***	22.55	$[M + NH_4]^+$	426.24749	$C_{22}H_{36}O_7N$	−2.672	MS^2[426.25]: 409.22077, **391.21046**, **373.19947**, 349.20030, **285.18457**; MS^3[391.21]: **331.18959**, **313.17888**, **295.16872**.	C	0.76
**50** **	22.94	$[M + NH_4]^+$	368.24223	$C_{20}H_{34}O_5N$	−2.497	MS^2[368.24]: **351.21578**, **297.18388**, 287.19949, 269.18937; MS^3[351.21]: **333.20457**, **315.19500**.	C	0.69
**51** **	24.98	$[M + NH_4]^+$	410.25300	$C_{22}H_{36}O_6N$	−1.741	MS^2[410.25]: 393.22627, **375.21587**, **357.20543**, 333.20470, **269.18925**; MS^3[375.22]: **357.20540**, **315.19482**, **297.18432**, **279.17401**, **251.17931**.	C	100.00
**52** *	25.94	$[M + NH_4]^+$	352.24734	$C_{20}H_{34}O_4N$	−2.541	MS^2[352.25]: **335.22070**, **317.21017**; MS^3[317.21]: **299.19965**, **281.18921**, **271.20486**, **253.19402**.	C	30.28
**53** **	26.38	$[M + NH_4]^+$	368.24235	$C_{20}H_{34}O_5N$	−2.171	MS^2[368.24]: **351.21581**, **333.20504**; MS^3[333.21]: **315.19534**, **297.18381**.	C	4.58
**54** *	26.54	$[M + NH_4]^+$	412.26825	$C_{22}H_{38}O_6N$	−2.703	MS^2[412.27]: **395.24252**, **377.23126**, **359.22076**, 333.24158, 315.23108, 273.22086, 255.20996; MS^3[359.22]: **341.21021**, **317.21024**, **299.19968**, **281.18918**, **271.20483**, **253.19429**, 225.16298, 197.13176, 169.10049.	C	27.33
**55** ***	26.82	$[M + NH_4]^+$	354.26331	$C_{20}H_{36}O_4N$	−1.623	MS^2[354.26]: **337.23686**, **319.22630**, 255.21036, 227.17892; MS^3[319.23]: **301.21562**, **283.20509**.	C	4.05
**56** ***	27.17	$[M + NH_4]^+$	470.27329	$C_{24}H_{40}O_8N$	−3.304	MS^2[470.27]: **453.24699**, **393.22615**, **333.20528**, **315.19485**.	C	1.17
**57** *	27.27	$[M + H]^+$	393.22580	$C_{22}H_{33}O_6$	−3.472	MS^2[393.23]: **375.21652, 333.20602**; MS^3[375.22]: **357.20593**, **333.20541**, **315.19522**, **297.18463**, **279.17404**, **269.18973**, **267.17410**, 253.15826, **251.17911**.	C	0.82
**58** ***	27.37	$[M + NH_4]^+$	454.27852	$C_{24}H_{40}O_7N$	−3.102	MS^2[454.28]: **437.25237**, **419.24142**, **377.23184**, **359.22109**, **317.21100**, **299.20005**, **281.18955**.	C	4.34

**59** **	27.88	$[M + NH_4]^+$	410.25263	$C_{22}H_{36}O_6N$	−2.643	MS^2[410.25]: **375.21556**, **315.19500**; MS^3[375.22]: **357.20546**, 339.19488, **297.18429**, **279.17389**.	C	0.85
**60** **	29.06	$[M + NH_4]^+$	410.25248	$C_{22}H_{36}O_6N$	−3.009	MS^2[410.25]: **375.21553**, 333.20547; MS^3[375.22]: **357.20534**, 339.19485, **315.19494**, **297.18435**, **279.17379**, **269.18943**, **251.17881**.	C	9.96
**61** ***	29.44	$[M + NH_4]^+$	398.25248	$C_{21}H_{36}O_6N$	−3.099	MS^2[393.23]: **381.22606**, **363.21569**, **345.20565**, 303.19482, 285.18383, 267.17331; MS^3[363.22]: **331.18940**.	C	0.79
**62** **	29.98	$[M + H]^+$	351.21381	$C_{20}H_{31}O_5$	−2.791	MS^2[351.21]: **333.20367**, **315.19324**, 305.20880, 287.19843, 269.18741, 251.17775; MS^3[333.20]: **297.18295**, **279.17239**.	C	0.79
**63** ***	32.20	$[M + NH_4]^+$	484.28895	$C_{25}H_{42}O_8N$	−3.188	MS^2[484.29]: **467.26253**, **449.25191**, 421.25843.	C	1.87
**64** *	33.20	$[M + NH_4]^+$	452.26346	$C_{24}H_{38}O_7N$	−1.811	MS^2[452.26]: **435.23743**, **417.22687**; MS^3[417.23]: **375.21652**, **357.20592**, **339.19460**, **329.21146**, **315.19460**, **297.18420**, **279.17360**, **269.19019**, **267.17370**, **251.17940**.	C	112.95
**65** **	34.44	$[M + NH_4]^+$	394.25592	$C_{22}H_{36}O_5N$	−2.880	MS^2[394.26]: **377.23016**, **359.21915**; MS^3[359.22]: **317.20892**, **299.19861**, 281.18805, 271.20410.	C	1.13
**66** **	34.91	$[M + NH_4]^+$	410.2526	$C_{22}H_{36}O_6N$	−2.716	MS^2[410.25]: 393.22615, **375.21566**, 287.19980, 285.18426, 267.17361, **251.17864**, 241.12156, 223.11112; MS^3[375.22]: **357.20534**, **315.19497**, **297.18441**, **279.17376**, **269.18974**.	C	43.67
**67** **	35.51	$[M + NH_4]^+$	412.26826	$C_{22}H_{38}O_6N$	−2.675	MS^2[412.27]: **395.24182**, **377.23157**, **317.21039**, **281.18854**; MS^3[317.21]: **299.19983**.	C	5.31
**68** **	36.50	$[M + NH_4]^+$	452.26065	$C_{24}H_{38}O_7N$	−3.629	MS^2[452.26]: **435.23636**, **375.21371**, 251.17887; MS^3[375.22]: **357.20522**, **315.19531**, **297.18426**.	C	61.69
**69** **	39.50	$[M + NH_4]^+$	452.26309	$C_{24}H_{38}O_7N$	−2.629	MS^2[452.26]: **435.23674**; MS^3[435.23]: **417.22682**, **375.21566**, **357.20528**, 339.19448, **315.19466**, **297.18423**, **279.17355**.	C	17.04
**70** ***	39.66	$[M + H]^+$	333.20364	$C_{20}H_{29}O_4$	−2.396	MS^2[333.20]: **315.19330**, 251.17793; MS^3[315.19]: **297.18304**, 287.19864, 269.18814.	C	3.83
**71** ***	40.42	$[M + H]^+$	303.23123	$C_{20}H_{31}O_2$	−2.067	MS^2[303.23]: **285.22079**, **267.21014**, 257.22599; MS^3[267.21]: **239.17843**, **225.16322**, **211.14779**, **197.13196**.	C	0.89
**72** **	41.55	$[M + H]^+$	435.23649	$C_{24}H_{35}O_7$	−2.849	MS^2[435.24]: **417.22607**; MS^3[417.23]: **357.20556**, 327.19503, **297.18494**, **279.17379**, 267.17373.	C	1.02
**73** ***	40.48	$[M + NH_4]^+$	454.27882	$C_{24}H_{40}O_7N$	−2.441	MS^2[454.28]: **437.25179**, **419.24145**, **359.22086**.	C	0.58
**74** *	41.00	$[M + H]^+$	435.23727	$C_{24}H_{35}O_7$	−1.057	MS^2[435.24]: **417.22702**; MS^3[417.23]: **375.21594**, **357.20505**, **339.19467**, **327.19464**, **315.19461**, **297.18417**, **279.17363**, **269.18918**, **267.17370**, **251.17890**.	C	17.27

**75** **	41.36	$[M + NH_4]^+$	352.24737	$C_{20}H_{34}O_4N$	−2.456	MS^2[352.25]: **335.22095**, **317.21042**, **271.20461**, 263.17838, 229.12130, 199.11118, 171.11637, 113.09562, 101.09568; MS^3[317.21]: **299.19977**, **281.18995**, **253.19451**, 217.12165, 215.10611, 203.10647.	C	6.62
**76** ***	41.64	$[M + NH_4]^+$	366.22662	$C_{20}H_{32}O_5N$	−2.402	MS^2[366.22]: 349.20027, **331.18977**; MS^3[331.19]: **313.17903**, **295.16782**, 267.17386.	C	1.62
**77** **	42.23	$[M + NH_4]^+$	452.26306	$C_{24}H_{38}O_7N$	−2.695	MS^2[452.16]: **435.23665**, **375.21587**, **357.20531**, 285.18411, **279.17389**, 267.17364, 241.12099, 223.11104; MS^3[375.23]: **315.19473**, **297.18417**.	C	13.72
**78** ***	42.77	$[M + NH_4]^+$	354.26318	$C_{20}H_{36}O_4N$	−1.990	MS^2[354.26]: **337.23634**, **319.22593**, 273.22046; MS^3[319.22]: **301.21605**, **283.20509**.	C	0.41
**79** ***	44.14	$[M + NH_4]^+$	496.28898	$C_{26}H_{42}O_8N$	−3.050	MS^2[496.29]: **479.26233**, **461.25216**, **419.24148**, **401.23123**, **359.22055**, 341.21048, 299.19959; MS^3[359.22]: 281.18928, 253.19407.	C	9.41
**80** ***	44.66	$[M + NH_4]^+$	350.23182	$C_{20}H_{32}O_4N$	−2.184	MS^2[350.23]: **333.20544**; MS^3[333.21]: **315.19488**, **297.18478**.	C	2.76
**81** ***	44.87	$[M + NH_4]^+$	366.22671	$C_{20}H_{32}O_5N$	−2.156	MS^2[366.22]: 349.20021, **331.18974**; MS^3[331.19]: **313.17924**, **295.16798**, 285.18432, 267.17367, 239.17890.	C	20.89
**82** ***	45.25	$[M + NH_4]^+$	392.24216	$C_{22}H_{34}O_5N$	−2.523	MS^2[392.24]: **375.21563**; MS^3[375.22]: **357.20531**, **333.20544**, **315.19488**, **297.18429**, **279.17373**, **269.18937**, 267.17437, **251.17884**.	C	7.77
**83** **	46.26	$[M + NH_4]^+$	368.24235	$C_{20}H_{34}O_5N$	−2.171	MS^2[368.24]: **351.21593**, **333.20544**; MS^3[333.21]: **315.19482**, 287.19812.	C	0.77
**84** **	46.72	$[M + NH_4]^+$	352.24746	$C_{20}H_{34}O_4N$	−2.200	MS^2[352.25]: **335.22110**, **317.21064**; MS^3[317.21]: **299.20005**, **281.18915**, **271.20583**.	C	0.72
**85** ***	47.00	$[M + NH_4]^+$	450.24743	$C_{24}H_{36}O_7N$	−2.663	MS^2[450.25]: **433.22113**; MS^3[433.22]: **391.21061**, **373.20002**, **355.18939**, 345.20470, **331.18965**, **313.17943**, **295.16887**, **277.15749**, 267.17358.	C	7.23
**86** ***	48.31	$[M + NH_4]^+$	496.28901	$C_{26}H_{42}O_8N$	−2.990	MS^2[496.29]: **479.26265**, **461.25216**, **401.23098**, **377.23126**, **317.21039**; MS^3[461.25]: **419.24182**, **359.22113**, **341.21027**, **299.19983**, **281.18928**, 263.17847, 253.19382.	C	36.42
**87** ***	48.64	$[M + H]^+$	421.25696	$C_{24}H_{37}O_6$	−3.573	MS^2[421.25]: **403.24644**, **361.23667**, **343.22547**, **283.20461**; MS^3[403.24]: **265.19429**.	C	1.72
**88** ***	50.46	$[M + NH_4]^+$	496.28953	$C_{26}H_{42}O_8N$	−1.942	MS^2[496.29]: **479.26210**, **461.25216**, **419.24179**, **401.23119**, **359.22073**.	C	1.01
**89** ***	52.08	$[M + NH_4]^+$	408.23703	$C_{22}H_{34}O_6N$	−2.533	MS^2[408.24]: **373.20011**; MS^3[373.20]: **355.18977**, 337.17918, **331.18980**, **313.17930**, **295.16878**, **277.15831**, **267.17386**, **249.16344**.	C	4.93
**90** ***	52.86	$[M + H]^+$	479.26214	$C_{26}H_{39}O_8$	−3.765	MS^2[479.26]: **461.25185**; MS^3[461.25]: **401.23077**, **341.21002**, **323.20026**, **281.18915**,	C	1.64
**91** ***	52.93	$[M + H]^+$	771.46547	$C_{44}H_{67}O_{11}$	−3.006	MS^2[771.46]: **753.45439**, **735.44527**, **717.43462**, 699.42336, **675.42416**, **657.41339**, 621.39294, **597.39227**, **579.38236**, **561.37219**, 551.38612, 543.35929; MS^3[657.41]: **639.40251**, **393.22617**.	C	1.49
**92** ***	54.29	$[M + H]^+$	785.44552	$C_{44}H_{65}O_{12}$	−1.953	MS^2[785.44]: **767.43420**, **707.41333**, **671.39234**, **661.40834**, **643.39745**, 565.36538, **375.21602**, **315.19476**, **297.18395**; MS^3[767.43]: **749.42453**, 731.41192, **689.40134**,	C	4.15

						653.38083, 635.36982, 625.38765, 611.37257, 593.36259, 575.35048, 557.33830, 547.35516, 529.34390, **393.22684**, **279.17331**.		
**93** **	55.09	$[M + NH_4]^+$	394.25623	$C_{22}H_{36}O_5N$	−2.570	MS^2[394.26]: 377.23125, **359.21933**, **299.19839**; MS^3[299.20]: 281.18835.	C	0.31
**94** ***	56.96	$[M + NH_4]^+$	538.2992	$C_{28}H_{44}O_9N$	−3.452	MS^2[538.30]: **461.25213**, 443.24197, 383.22064, 323.19998; MS^3[461.25]: **419.24093**, **401.23022**, **359.22049**, **341.21008**, **299.19983**, **281.18912**, 263.17844.	C	10.96

Notes: *Confirmed by comparing with the reference compounds; **compounds have been reported in literatures; ***potential new ent-kaurane diterpenoids. In the “type” column, A denotes bridgehead-unsubstituted 7,20-epoxy-ent-kaurane diterpenoids; B denotes bridgehead-substituted 7,20-epoxy-ent-kaurane diterpenoids; and C denotes 7,20-non-epoxy-kaurane diterpenoids. The bolded fragment ions are the key diagnostic ions for each compound. "Rel. Abund." denotes relative abundance, calculated as the percentage of a compound's peak area relative to the maximum peak area in the TIC.
